# A century of changing flows: Forest management changed flow magnitudes and warming advanced the timing of flow in a southwestern US river

**DOI:** 10.1371/journal.pone.0187875

**Published:** 2017-11-27

**Authors:** Marcos D. Robles, Dale S. Turner, Jeanmarie A. Haney

**Affiliations:** The Nature Conservancy, Center for Science and Public Policy, Tucson, Arizona, United States of America; Oregon State University, UNITED STATES

## Abstract

The continued provision of water from rivers in the southwestern United States to downstream cities, natural communities and species is at risk due to higher temperatures and drought conditions in recent decades. Snowpack and snowfall levels have declined, snowmelt and peak spring flows are arriving earlier, and summer flows have declined. Concurrent to climate change and variation, a century of fire suppression has resulted in dramatic changes to forest conditions, and yet, few studies have focused on determining the degree to which changing forests have altered flows. In this study, we evaluated changes in flow, climate, and forest conditions in the Salt River in central Arizona from 1914–2012 to compare and evaluate the effects of changing forest conditions and temperatures on flows. After using linear regression models to remove the influence of precipitation and temperature, we estimated that annual flows declined by 8–29% from 1914–1963, coincident with a 2-fold increase in basal area, a 2-3-fold increase in canopy cover, and at least a 10-fold increase in forest density within ponderosa pine forests. Streamflow volumes declined by 37–56% in summer and fall months during this period. Declines in climate-adjusted flows reversed at mid-century when spring and annual flows increased by 10–31% from 1964–2012, perhaps due to more winter rainfall. Additionally, peak spring flows occurred about 12 days earlier in this period than in the previous period, coincident with winter and spring temperatures that increased by 1–2°C. While uncertainties remain, this study adds to the knowledge gained in other regions that forest change has had effects on flow that were on par with climate variability and, in the case of mid-century declines, well before the influence of anthropogenic warming. Current large-scale forest restoration projects hold some promise of recovering seasonal flows.

## Introduction

Mountain environments provide about half of the world’s population with water [1], and this reliance is particularly acute in semi-arid regions including the southwestern United States (US) where both human and natural communities rely heavily on freshwater derived from upland forests. This region is vulnerable to current and projected shortages in water supply [2], a concern that has been heightened by recent changes in snowpack, which stores water in winter months and delivers it in spring and summer months. A recent study in the Sierra Nevada, for example, found snowpack levels at a 500-year low [3]. Broader regional studies have documented that in the last five decades snowpacks across western US mountain ranges have declined by about 20% [4–6], the fraction of snowfall versus rainfall has declined [7], and peak spring flows are occurring earlier [8–11].

To date, annual flow magnitudes have not consistently declined across the region in response to these changes in snowpack levels [8,12–15]. However, flow timing has been altered with increased flow in winter and early spring months and decreased flow in summer months [9,11,14,16]. Studies have found that roughly half of the observed changes in hydrology since mid-century were the direct result of warming due to human activities, with the remaining half due to drought conditions [5,17,18]. Whereas precipitation in the region fluctuated between wet and dry periods [13,19,20], temperature change has been identified as the persistent change agent that will cause future declines in snowpack and streamflow levels as well as earlier timing of spring runoff [21,22].

Much less attention has been paid to forest effects on streamflow, although foundational research in the region and globally shows that reductions in forest cover and basal area can lead to increases in streamflow, and conversely, reforestation or afforestation practices have been associated with flow declines [23–30]. Restoration projects that reduce forest basal area and canopy cover can influence flows by decreasing evapotranspiration losses [31] and increasing snowpack accumulation [32]. Studies that use statistical and modeling approaches to compare the relative influences of climate *and* land use change on flows have found that in many watersheds, the effects of land use change, including reforestation, can have similar magnitudes to and counteract the effects of climate variability [33–36]. To our knowledge none of this research has been conducted in the southwestern US.

The magnitude and duration of flow alterations associated with forest change can vary considerably due to regional climate, management practices, and local factors such as watershed characteristics, soils, forest types, vegetation types, and wildfire history [12,27,33,37–39]. Therefore, several unique characteristics need to be considered to evaluate streamflow trends in the focal area of this study, central Arizona in the southwestern US. First, streamflow levels in this region are sensitive to changes in vegetation cover because approximately 90% of incoming precipitation is lost to evaporative demand and transpiration by vegetation [39–41]. Second, measurements across many regional forest stands demonstrate that current densities and basal areas of forests are significantly greater than historical conditions due to fire suppression [42–47]. Third, the hydrologic regime is intermediate between snow-dominated climates in the Northern Rocky and Sierra mountains and precipitation-dominated mountains along coastal California, Oregon and Washington [9]. Snow only accumulates at the highest elevations in this region, runoff and flooding from winter rainfall events are not uncommon [48], and the North American Monsoon accounts for about half of annual precipitation [49] and has a significant influence on summer flows. These characteristics and high inter-annual variability in precipitation [13] may explain why watersheds in Arizona and New Mexico did not consistently follow trends of recent snowpack declines and earlier timing of spring flows reported elsewhere in the West [4,11].

**Fig 1:**
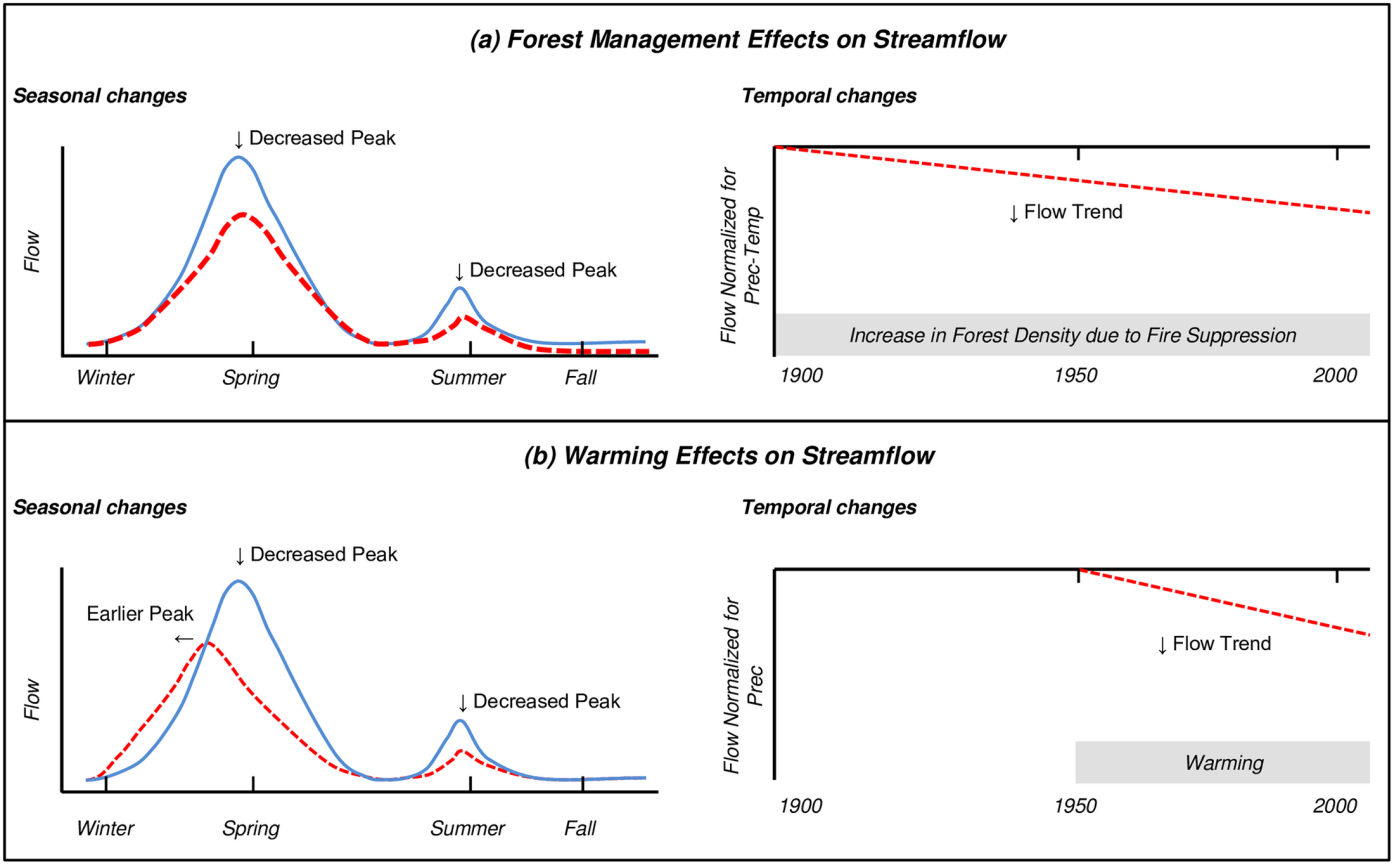
Study hypotheses. Seasonal and temporal changes to 20^th^ century streamflow in the Salt River, Arizona that were hypothesized to be the result of (a) forest management and (b) warming. Increases in forest density due to forest management policy of fire suppression [44] assumed to occur across full century. Increases in temperature due to human activities assumed to affect hydrological processes since mid-century [5,17,18,51]. Blue solid lines represent unaltered flow regimes whereas red dashed lines represent altered flow regimes. Left panels show seasonal changes; right panels show temporal departures from flows normalized for climate variability.

The purpose of this study was to evaluate trends and drivers of streamflow in the Salt River in the 20^th^ century. The Salt River is an important regional river because it, along with its tributary Verde River, provides up to 40% of the water supply for the Phoenix Metropolitan Area [50]. We hypothesized that absent any directional change in precipitation over the last century [13], changes in seasonal flow, if they occurred, would be due to increases in forest density from a century of fire suppression and/or a half-century of rising temperatures due to human activities (Fig 1). Specifically, we hypothesized that increases in the forest densities and basal areas would be associated with declines in spring and summer flows and that anthropogenic warming beginning at mid-century [51] would be coincident with earlier peak spring flows and declines in late spring and summer flows. Hypotheses were based on aforementioned link between warmer temperatures and hydrologic change [4,5,7,9–12,52], streamflow declines coincident with increases in forest density [44,53,54], and research that evapotranspiration losses are minimized [31] and snowpack levels are enhanced [32,55] in forests with intermediate densities relative to high-density forests. In order to increase the power to detect time trends in streamflow which is highly variable in the watershed [13], we adapted a hydrologic procedure that first accounts for the relationship of flows to climate parameters using linear regression models, and then removes climate effects by testing for time trends in the residual flows from these models [56].

## Materials and methods

### Study area

**Fig 2:**
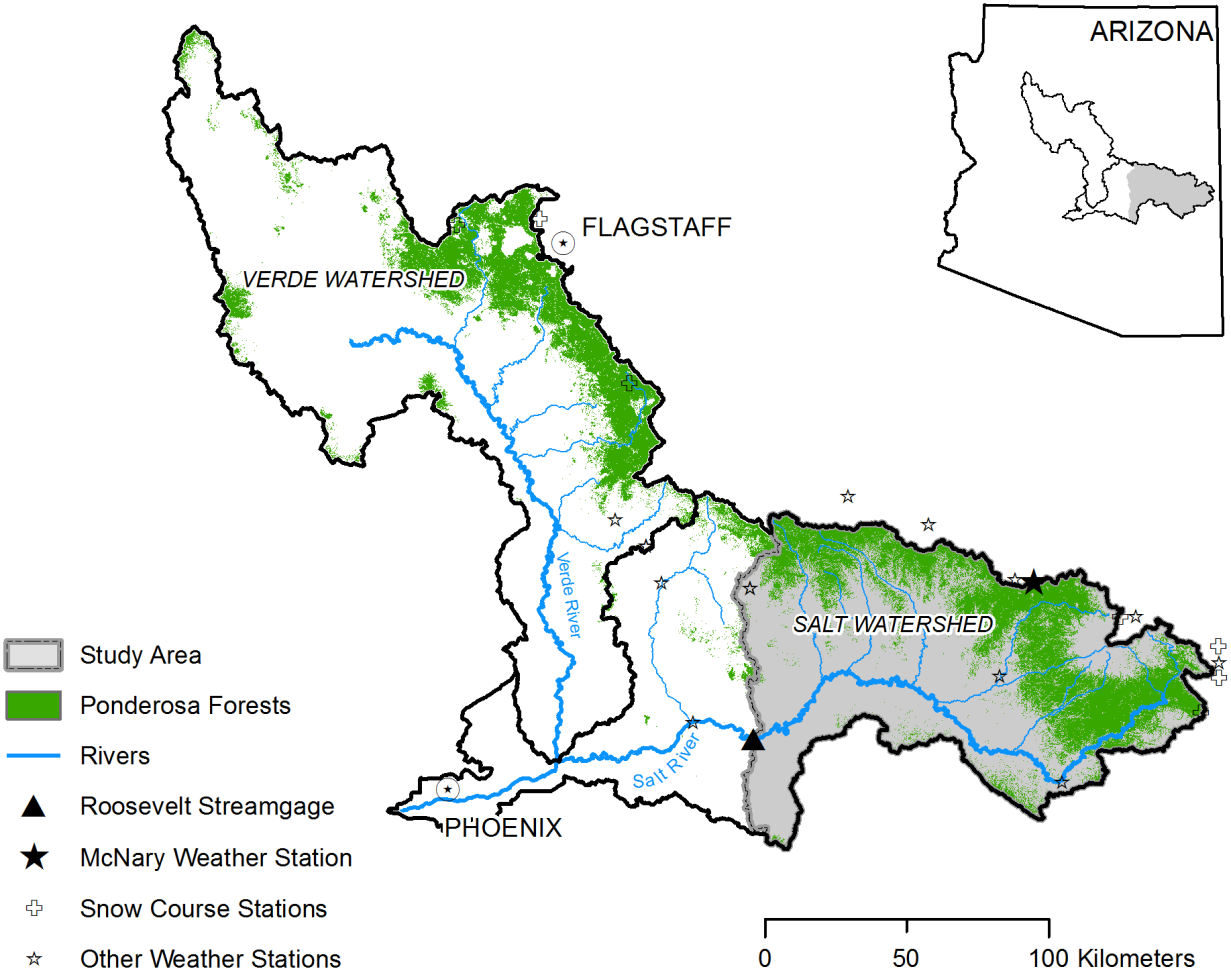
Study area map. Map showing the study watershed in grey along with neighboring portions of Salt and Verde watersheds in central Arizona [58]. Extent of ponderosa pine forests that store snowpack in winter months and are a major source of streamflow shown in green, from US Geological Survey Gap Analysis Program [59]. Locations of study flow gage, US Geological Survey streamgage Salt River near Roosevelt (#09498500) [60], National Weather Service climate station near McNary (NWS Station #025412) [61] and other stations [61,62] considered are also indicated.

The Salt River watershed above Roosevelt Lake covers 11,108 km^2^ (2.74 million acres) in central Arizona (Fig 2). The primary watershed managers are the White Mountain Apache Tribe (61% of the land area, 74% of the ponderosa pine area), US Forest Service (26% of land, 21% of ponderosa), and San Carlos Apache Tribe (11% of land, 5% of ponderosa). Elevations range from 640 m to 3,476 m (2,177–11,403 ft), with mountains in the high northern and eastern portions of the watershed, deeply incised streams with steep side slopes in the middle-elevation central parts, and more moderate side slopes and plateaus in the low-elevation southern and western areas. Soils are derived from basalt (47%) and sedimentary rock (53%) [57].

From high to low elevations, vegetation types in the watershed include: mixed conifer forests, ponderosa pine forests, mountain grasslands, pinyon-juniper woodlands, chaparral shrublands, and mesquite scrub. Forests occur at elevations above 1,524 m (5,000 ft) and in areas with greater than 508 mm (20 in) of annual precipitation. Mixed conifer forests grow at the highest elevations and occupy 7% of the watershed area [59]. Ponderosa forests grow across 30% of the watershed at mid to high elevations, from 1,800 to 2,600 m (5,905–8,530 ft) [59,63]. Ponderosa pine trees are dominant but forests include species of fir, aspen, other pines, and oaks. Riparian areas in the bottom of the stream valleys have a large diversity of vegetation that varies with elevation [64].

### Climate and hydrology

The climate is semiarid across most of the study area, transitioning to a dry sub-humid climate at the highest elevations where precipitation exceeds evapotranspiration [65]. Over the full study period (1914–2012), mean annual precipitation in the Salt River basin above Roosevelt averaged 575 mm (23 in), ranged from 302 to 957 mm across years (11–38 in) and 380 mm to 890 mm across elevations (15–35 in) [66]. At elevations above 2,000 m (6,600 ft), precipitation falls primarily as snow from November through March. Mean annual snowfall ranged from 1,270 mm (50 in) at lower elevations to 2,290 mm (90 in) at high elevations. Though occupying only a small fraction of the watershed, elevations above 2,743 m (9,000 ft) can experience annual snowfall as high as 5,080 mm (200 in) [61].

**Fig 3:**
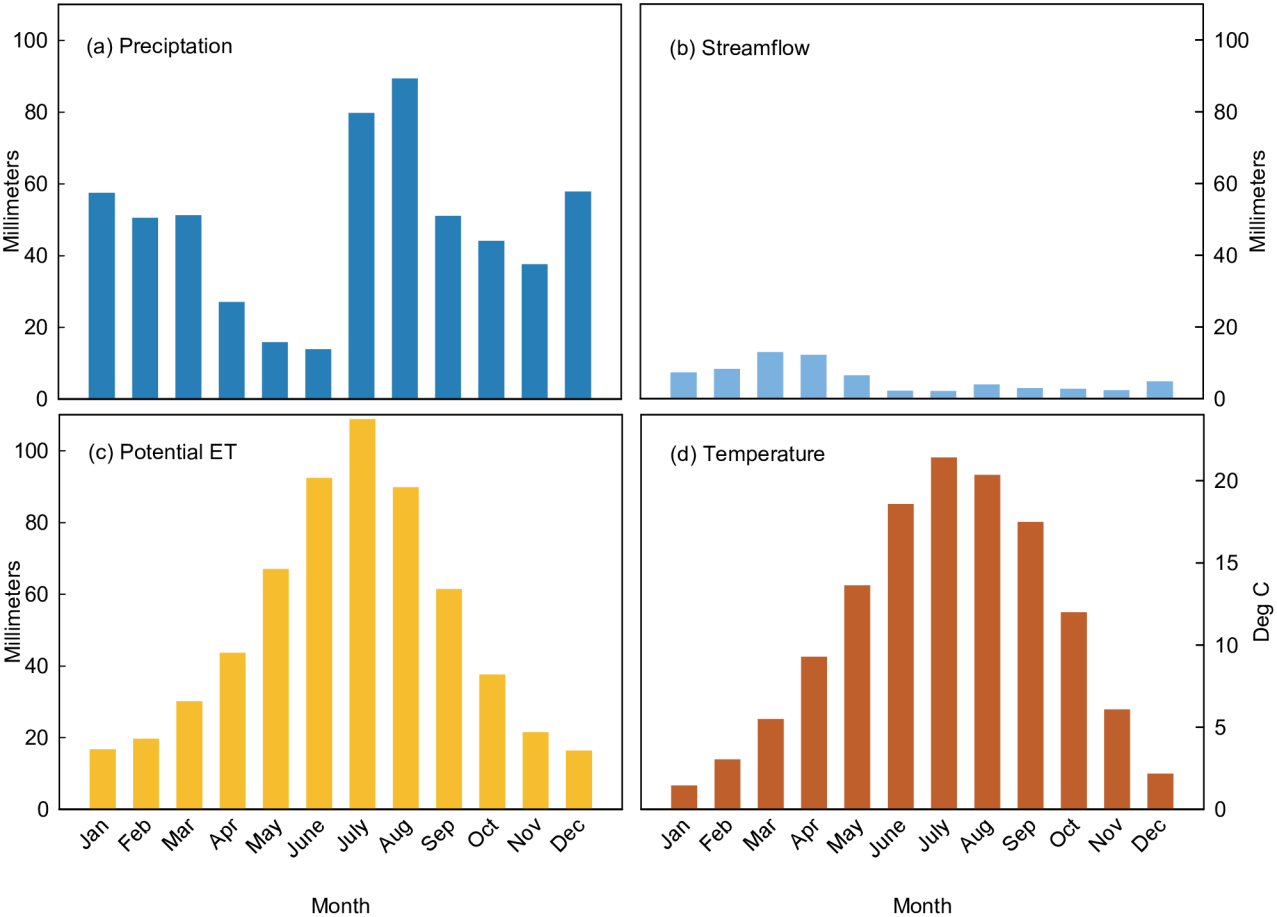
Flow and climate characteristics. Monthly averages from 1914–2012 of (a) precipitation, (b) streamflow, (c) potential evapotranspiration, and (d) temperature and in the Salt River watershed in central Arizona. Streamflow data from US Geological Survey stream gage Salt River near Roosevelt (USGS station 09498500) [60]. All other parameters derived from PRISM model [66] clipped to study boundary.

Precipitation has a bimodal distribution where mid-latitude frontal storms deliver moisture from November to March from the Pacific Ocean and the North American Monsoon delivers moisture from July to September from the Gulf of Mexico and California (Fig 3). Winter storms are long-duration, low-intensity and extend over large areas, whereas summer thunderstorms are short-duration, high-intensity, and occur with high spatial variability. While most of precipitation from winter storms falls as snow at mid to high elevations, heavy rainfall events in this season can produce the largest runoff events in a year [48]. Fall months (late September to November) and late spring to early summer months (April to June) are transitional dry periods, though rainfall levels in fall months can occasionally be high due to early frontal systems or dissipating tropical storms.

Mean annual temperature over the full study period was 10.9°C (52°F); annual means ranged from 9.8°C (50°F) to 12.5°C (55°F) [66]. High temperatures drive large evapotranspiration losses with the result that streamflow was only about 10% of incoming precipitation (Fig 3). Potential evapotranspiration peaks in summer months when the demand across the watershed exceeds supply [66,67]. Actual evapotranspiration in forests is limited by available water supply and thus peaks slightly later, in August to September [68], when monsoon rains replenish soil moisture that has been depleted in the dry summer months of May and June.

Mean annual streamflow of the Salt River near Roosevelt was about 776 million m^3^ (630,000 acre-ft) over the full study period [60]. Following the precipitation regime, the distribution of streamflow is bimodal with roughly 80% of streamflow produced in winter to spring months (December to May) and a smaller peak occurring in the summer months associated with summer monsoon rainfall (Fig 3). With annual base flow of 208 million m^3^ (169,000 acre-feet), groundwater discharge was estimated to be 27% of average annual streamflow [69]. Regional bedrock aquifers discharge groundwater into the Salt River, producing relatively constant annual base flow where inter-annual variation is dampened.

### Study hypotheses and flow regimes

We developed hypotheses that predicted forest and temperature effects on historical seasonal streamflow in the Salt watershed based on a regional literature review (Table 1). The hypotheses described the expected direction of influence that temperature and forest change would have on flows, the seasons and time periods when the influence was most likely to be apparent, and changes to intermediate mechanisms, such as evapotranspiration and sublimation, that would directly bring about the flow changes induced by temperature or forest change. Although we did not directly investigate these mechanisms in this study, their relationships with seasonal flows and climate were well established in the literature as referenced in Table 1.

**Table 1:** Detailed study hypotheses.

	*Expected Period Trends*		*Expected Seasonal Trends*				*References*
*Description*	*1914–1963*	*1964–2012*	*Win*	*Spr*	*Sum*	*Fall*	
1. An *increase in forest density* has led to increases in evapotranspiration losses and decreases in soil moisture, producing a *decrease in summer and fall flows*.	↓	↓			↓	↓	[28,29,31,43–47,53,70–74]
2. A *decline in size and number of forest canopy openings* has led to an increase in tree canopy interception and sublimation losses, resulting in snow pack declines and subsequent *reductions in spring flows*.	↓	↓		↓			[32,55,75,76]
3. *Increased temperatures* have led to earlier peak spring flows and increased evapotranspiration losses from forests, soils and streams, producing a *decrease in summer and fall flows*.		↓			↓	↓	[14,52,77]
4. *Increased temperatures* have led to an increase in snow sublimation vapor losses, resulting in snowpack declines and subsequent *reductions in spring flows*.		↓		↓			[4–6,8,10,14,18,78]
5. *Increased temperatures* have increased the energy available for snow melt leading to earlier peak spring snowmelt and *earlier peak spring flows*.		Δ Flows		Δ Flows			[8–11,79,80]

Hypotheses that describe the anticipated cause-effect relationship between forest and climate change, intermediate mechanisms, and streamflow based on regional literature review. Symbols in columns describe predicted declines (downward arrow) or change in flow timing (Δ flows) within indicated study time-periods and seasons.

We also developed conceptual flow regimes that described our assumptions on how climate and seasonal water storage patterns would influence monthly flows (Table 2). We categorized monthly flow into three regime types—spring snowmelt, summer monsoon and winter baseflow—based upon climate, storage and flow relationships studied in previous ecohydrological studies conducted within southwestern ponderosa pine forests [41,68,72,81], climatological studies within or near the Salt River watershed [48,67,82–84], and a groundwater model developed for northern Arizona [69]. We assumed that the two primary storage pools influenced by precipitation were snowpack and soil moisture, while evapotranspiration and sublimation were the primary mechanisms by which temperatures would influence flows. Changes in groundwater storage could also influence flows but we expected these changes to be spread out over years, not seasons, because the underlying regional aquifer is large and the high elevation recharge areas are distant from downstream discharge areas. A groundwater model simulation in the basin found that the range of variation in base flows from groundwater discharge was lower than the range of variation in recharge rates [69], suggesting that the groundwater system is relatively buffered from climatic variability.

**Table 2:** Conceptual flow regimes.

*Flow Regime*	*Month*	*Precipitation influence*				*Temperature influence*			
		*Antecedent Months*	*R*	S_SM_	*S*_*SP*_	*Antecedent Months*	*ET*	*S*_*S*_	*SM*
Spring Snowmelt	Jan	Nov–Jan	X	X	X	Nov–Jan		X	X
	Feb	Nov–Feb	X	X	X	Nov–Feb		X	X
	Mar	Nov–Mar	X	X	X	Nov–Mar		X	X
	Apr	Nov–Apr	X	X	X	Nov–Apr	X	X	X
	May	Nov–May	X	X	X	Nov–May	X	X	X
	Jun	Nov–Jun	X	X	X*	Nov–Jun	X	X*	X*
Summer Monsoon	Jul	Jan–Jul	X	X	X*	Jan–Jul	X	X*	X*
	Aug	Jul–Aug	X	X		Jul–Aug	X		
	Sep	Jul–Sep	X	X		Jul–Sep	X		
	Oct	Jul–Oct	X	X		Jul–Oct	X		
Winter Baseflow	Nov	Oct–Nov	X	X	X	Oct–Nov		X	X
	Dec	Oct–Dec	X	X	X	Oct–Dec		X	X

Assumed relationships between seasonal precipitation, temperature and monthly flows based on regional ecohydrology, climatology, and groundwater studies. Columns abbreviations are: R = Runoff; S_SM_ = Soil Moisture Storage; S_SP_ = Snowpack Storage; ET = Evapotranspiration; S_S_ = Snow Sublimation; SM = Snow Melt.

* symbol indicates anticipated relationship would only occur in extreme years with late snowfall/snowmelt.

### Data

#### Forest conditions and management

We collected data from several sources to provide a qualitative assessment of changes in forests conditions and fire throughout the 20^th^ century in or near the Salt River watershed (Table 3). We found data to compare historical (1867–1925) to current (2000–2012) forest conditions. Because few historical data were available directly within the study watershed, we used historical estimates from ponderosa pine sites in northern Arizona for canopy cover (n = 6) [85] and from the Southwest for basal area (n = 24) and stem density (n = 34) [86]. Basal area data from 1951–1952 were available on sites (n = 4) within the Salt River watershed [71]. Estimates of current forest basal area, canopy cover, and density were derived from sites in the watershed, summarized from US Forest Inventory and Analysis (FIA) program plots (n = 143, post-2000) [87,88]. We also summarized estimates of these parameters across the study watershed from the Integrated Landscape Assessment Project (ILAP), a continuous raster layer (30x30m pixels) of modeled data built with FIA plot data and 2006 Landsat imagery [89].

**Table 3:** Historical forest and human water demand.

Category	Type	Jurisdiction	Years with Data	Years with Data Gaps	Sources
Forest Management	Forest Density, Basal Area, Canopy Cover	US Forest Service	Historical (1867–1925) vs. Current (2000–2012)	Not Continuous	[85–89]
		Tribal	Historical (1951–1952); Current (2000–2012)	Not Continuous	[71,87–89]
Forest Management	Logging/Thinning	US Forest Service	1914–1962, 2006–2012	1963–2005	US Forest Service, unpublished
		Tribal	1918–1981	1914–1917; 1982–2012	[90]
Forest Management	Prescribed Fire	US Forest Service	2006–2012	1914–2005	[91]
		Tribal	1947–1971	1914–1946; 1972–2012	[90]
Forest Management	Wildfire	US Forest Service	1970–2012	1914–1969	[91,92]
		Tribal	1947–1971, 1984–2012	1914–1946; 1972–1983	[90,92]
Human Water Use	-	US Forest Service	1971–2008	1914–1970; 2009–2012	[50,93]
		Tribal	1971–2008	1914–1970; 2009–2012	[50,93]

Summary of years with available data, including data sources, for forest management and human water demand activities in or near Salt River watershed in central Arizona from 1912–2012.

To derive logging estimates, we used timber inventory and sales data available from the White Mountain Apache Reservation from 1918–1981 [90] and the US Forest Service Alpine Ranger District and Pleasant Valley Ranger District from 1910–1959 and 1934–1962, respectively (data unpublished). These jurisdictions represented 74%, 12%, and 5% of the ponderosa pine forests in the study watershed, respectively. All timber sales were originally reported in volumes of wood harvested. US Forest Service inventories and sales were reported by Public Land Survey geographic units: township, range and section. To estimate the area affected by timber cutting, we calculated the mean inventoried volume per unit area for each legal section (259 ha/640 acres), producing estimates of 21.1 m^3^/ha (3,618 board-feet/ac) for the Pleasant Valley Ranger District and 26.2 m3/ha (4,485 board-feet/ac) for the Alpine Ranger District. The volumes of timber sales for each year were divided by these ratios to estimate area cut for each respective ranger district. Lacking comparable inventory data for the White Mountain Apache Reservation, we used the ratio from Alpine Ranger District to estimate area affected by timber cutting there because this district had a much larger sample size (301 versus 26 sections).

Records on prescribed fires were available for the White Mountain Apache Reservation from 1947–1971 [90] and from the US Forest Service from 2006–2012 [91]. Wildfire data for the watershed were available from 1947–1971 for the White Mountain Apache Reservation [90], from 1970–1983 for US Forest Service lands [91], and for both jurisdictions from 1984–2012 [92]. Some wildfire data were only available as multiyear totals and were annualized assuming an even distribution of area burned every year. Data on wildfires from 1984–2012 were derived from the Monitoring Trends in Burn Severity (MTBS) database clipped to ponderosa pine forests in the watershed [92].

#### Human water demand

Human water demand was low because the watershed is sparsely populated, with about 20,000 residents as of 2010 [94]. Data on residential, industrial and agricultural water uses in the Salt River watershed, including groundwater, surface water use and surface water diversions, were based on estimates for the Salt River watershed from 1971–2008 from the Arizona Water Atlas [50] and the Arizona Water Resources Development Commission [93] (Table 3).

#### Climate and streamflow

We extracted total monthly flow values from 1914–2012 from the US Geological Survey (USGS) Salt River streamgage near Roosevelt, Arizona (USGS #09485000) (Fig 2) [60]. For climate trend and correlation analyses, we used monthly total precipitation and monthly mean temperature data from the McNary cooperative weather station (National Weather Service NWS Station #025412) located in the study boundaries near Pinetop, AZ (Fig 2) [61]. To evaluate snowpack trends, we extracted Feb 1, Mar 1, and Apr 1 snow water equivalent (SWE) data from multiple snow course stations within or near the study watershed from 1964–2012 [62]. We also used climate data from the PRISM model (4km x 4km pixels) [66] clipped to the watershed boundary to conduct additional precipitation and temperature regressions analyses and to calculate monthly averages of precipitation, temperature and potential evapotranspiration (Hamon equation [95]).

The McNary cooperative station had > 90% of possible months with data and the highest correlation coefficients with total monthly flows of the 15 stations tested. Months that had more than five days of missing data were classified as months with no data. Data from McNary only went back to 1934, so we extended the record from 1914–1933 based on regressions with a nearby site, Whiteriver 1SW (NWS #029271), for common years 1941–2008 (for a detailed description of climate stations and extension of records, see S1 Text).

One climate site was used for all regression analyses so that monthly trends could be compared with data that had the same relations between climate and flows. At an elevation above 2,240 m (7,340 ft), the McNary station was likely representative of the higher elevation ponderosa pine forests in the watershed where snowpack accumulation contributes significantly to streamflow [28,29,39]. It was also likely representative of winter and spring precipitation events which typically extend over large areas, but we anticipated that relations between station precipitation data and flow would be less robust for summer months because monsoon rainfall has high spatial variability. For this reason, we re-ran the regression models of summer flow (July-September) using total monthly precipitation and mean monthly temperatures extracted from the PRISM model across the entire watershed area [66].

### Trend analyses

We analyzed trends in (a) monthly precipitation, temperature and snowpack; (b) annual and monthly streamflow; (c) annual and monthly streamflow with the effects of precipitation removed; (d) annual and monthly streamflow with the effects of precipitation and temperature removed; and (e) the timing of peak spring flows. Trends were evaluated across three time periods: the full streamflow record, 1914–2012, and two sub-periods, 1914–1963 and 1964–2012. The two sub-periods were selected to capture the overlapping and non-overlapping portions of forest management and warming influence (Fig 1, Table 1). For ease of presentation, we also refer to the full period as “20^th^ century” and the two sub-periods as the “1^st^” and “2^nd^ half” of the 20^th^ century. All trend analyses were done using the statistical software package SPLUS (TIBCO Software, Inc., Palo Alto CA) and SAS version 9.3 (SAS Institute, Inc., Cary NC). The threshold for significance level used for all tests was a p-value equal to or below 0.05. Tests with p-values between 0.05 and 0.10 were termed “nearly significant” as these levels also indicate a strong association.

The annual flow time step was evaluated to verify that water budgets changed over time given that we were unable to evaluate all water budget terms. We assumed that soil moisture [41] and snowpack storage in the Salt River watershed reach their lowest level at the end of June (distant from winter precipitation, before onset of monsoon), so we calculated annual streamflow trends using a July–June water year (1914 excluded from annual analysis because streamgage record began in January 1914). The monthly time step was chosen because we were interested in seasonal changes in flow associated with climate and other factors. Additionally, historical flow and climate data were consistently available only at the monthly scale.

A two-step procedure was used to increase statistical power for detection of time trends associated with forest and temperature effects by decreasing flow variance due primarily to precipitation [56]. First, we developed multiple linear regression models (described in next section) to account for climatic influences on streamflow, and then, we tested whether trends from model residuals were significant and consistent with the hypothesized season and direction of forest or temperature change effects (Fig 1, Table 1). Forest effects were evaluated indirectly using climate-adjusted flow trends because we did not have a complete time series of forest condition data to add as explanatory variables in models. Temperature effects were evaluated from precipitation model residuals and directly from precipitation-temperature models when temperature terms were significant. A limitation of this method is that residuals contain model error. However, we assumed that if residual trends were significant and in the same direction as the predicted influences of forest or temperature effects, that it was probable that the trend was due to these factors as there was no a priori reason to expect model errors would have a significant temporal trend or bias. Inferences on temperature and forest effects on residual trends were corroborated by a regional literature review presented in the discussion section.

The nonparametric Mann-Kendall test and Sen’s method for estimation of slopes were used to determine significance and magnitude of trends in flow and climate variables [56,96]. Additionally, the Regional Kendall test was used to assess trends in total monthly snowpack across multiple snow course sites in order to increase statistical power for trend detection [97]. These methods were considered appropriate because streamflow and precipitation data are not normally distributed and outliers can unduly influence parametric trend tests. To facilitate comparison and interpretation, we estimated magnitudes or volumes of flow changes across a given period by multiplying Sen’s slope coefficients by the number of years in the period evaluated and converted flow rates (m^3^/s) to volumes (million m^3^).

#### Multiple linear regressions

Before regression models were built, we assessed normality of each variable and plotted monthly flow against several candidate climate parameters to see if relationships were likely to meet assumption of linearity. Monthly flows were log transformed because absolute values had positively skewed distributions. Initial plots revealed a curvilinear relationship between precipitation and log-flow in months influenced by snowmelt (Table 2); therefore, a quadratic form of precipitation variables was used in regression models in these months to improve linear model fit.

We used a stepwise regression method to build models. The first precipitation variable tested was precipitation for the same month of streamflow, then antecedent months of precipitation were tested. Up to two precipitation terms that explained the most variation in monthly flows were first added. Then up to two temperature terms were added. As we built the models, we used the conceptual flow regimes (Table 2) as a point of reference from previous research findings, but tested precipitation and temperature terms up to one-year prior of monthly flow to avoid any bias. Consistent with previous research in the region showing that precipitation exerts a dominant influence on flows [28,29,39,98,99], we added precipitation terms first because initial trial regressions indicated that precipitation accounted for greater than 90% of streamflow variation explained.

Models were selected if they met the following criteria: (1) model explained most variation in streamflow (adjusted r^2^) with lowest standard error; (2) explanatory variables were significant at p-value less than or equal to 0.05 and not correlated with one another (Variance Inflation Factor (VIF) < 2.0); and (3) residuals were normally distributed and independent. We assessed serial correlation of residuals using the Mann-Kendall test on residuals versus lag-1 residuals [56]. This test revealed serial correlation for July, August and November flow models which we eliminated by removing every fourth year of data from these models [56]. Exceptions to these criteria were allowed for quadratic precipitation terms where, in some cases, the squared term was not significant and VIFs indicated correlation of precipitation terms. Because a suggested remedy for this situation, data centering of explanatory variables (value—mean) [56], lowered VIFs to acceptable levels (<2.0) and resulted in equivalent p-values and adjusted r^2^ values as models with un-centered terms, the final models had un-centered terms.

In total, four types of precipitation-temperature models were developed:

$$logQ\;=\;\beta_{\text{1}}P_{\text{1}}\;+\;\beta_{\text{2}}P_{\text{2}}+\;\beta_{\text{0}}+\;\varepsilon \;(Linear\;P)$$

$$logQ\;=\;\beta_{\text{1}}P_{\text{1}}\;+\;\beta_{\text{2}}P_{\text{2}}+\;\beta_{\text{3}}T_{\text{1}}+\beta_{\text{4}}T_{\text{2}}+\beta_{\text{0}}+\;\varepsilon \;(Linear\;P,\;T)$$

$$logQ\;=\;\beta_{\text{1}}P\;+\;\beta_{\text{2}}P^{\text{2}}+\beta_{\text{0}}+\;\varepsilon \;(Quadratic\;P)$$

$$logQ\;=\;\beta_{\text{1}}P\;+\;\beta_{\text{2}}P^{\text{2}}+\;\beta_{\text{3}}T_{\text{1}}+\beta_{\text{4}}T_{\text{2}}+\beta_{\text{0}}+\;\varepsilon \;(Quadratic\;P,\;T)$$

where

*logQ* = base 10 logarithm flow (m^3^/s)

*P*_*1-2*_ = precipitation terms (mm)

*P + P*^*2*^ = quadratic precipitation terms

*T*_*1-2*_ = temperature terms (°C)

*β*_*1–4*_ = slope coefficients

*β*_*0*_ = intercept

*ε* = error

#### Trends in spring flow timing

We modified a center of timing (CT) flow measurement to estimate the date at which 50% of the flows between January and June was reached. This metric, an indicator of peak spring snowmelt timing, is typically calculated across the entire water year (Oct-Sep) [8,9,11]. However, we observed several annual hydrographs in the study streamgage when flows were highest in summer months (e.g. presumably years with low winter and high monsoon precipitation). We concluded that CT calculated across the entire water year would skew toward later dates in these years for reasons associated with inter-annual variability in precipitation that would be unrelated to the hypothesized temperature effects. We chose the January-June time frame to exclude tropical storms that can lead to heavy rainfall (but not snow) in October to December and the summer monsoon which is most prevalent from July to September.

## Results

### Changes in forest conditions

**Fig 4:**
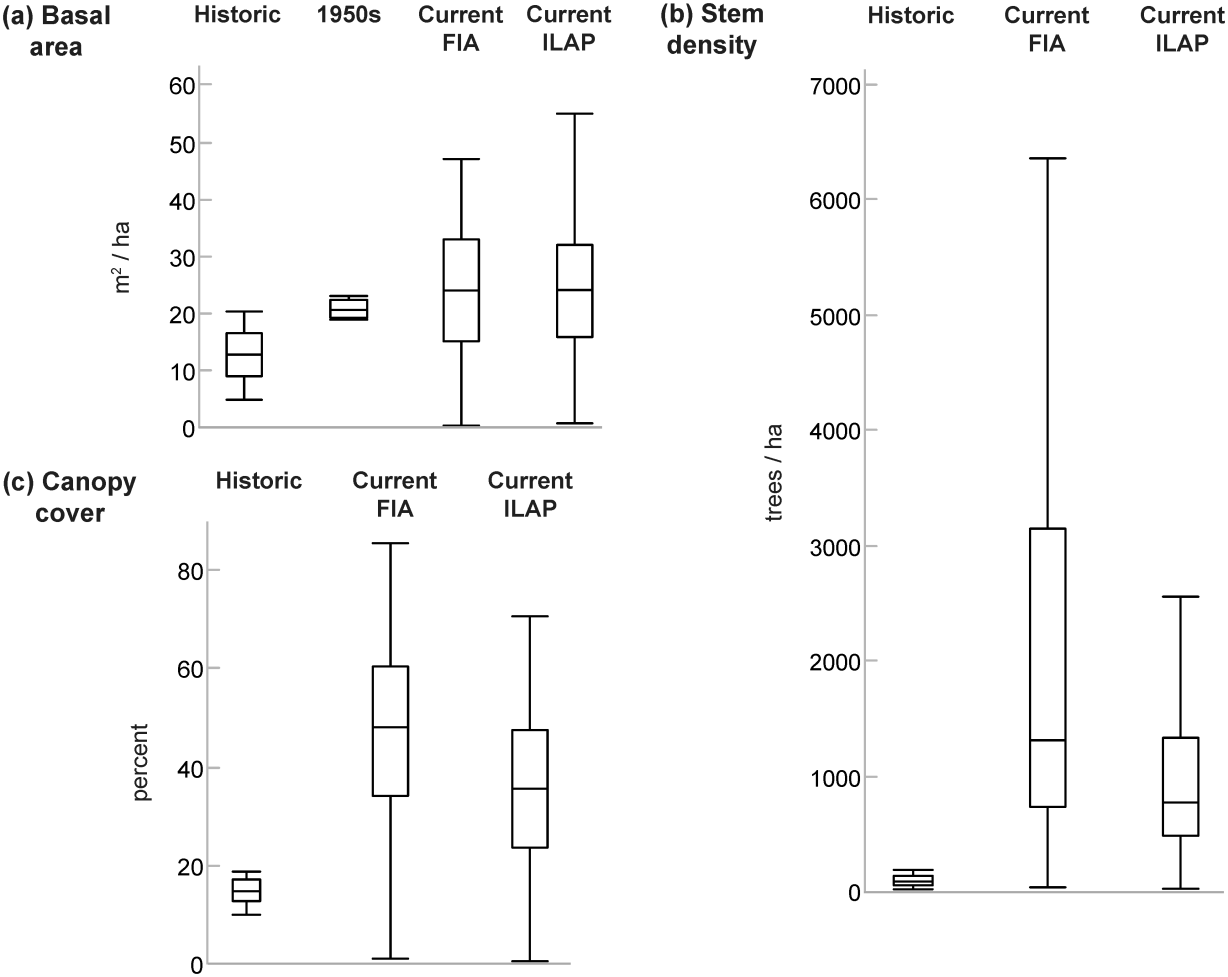
Historical changes in forest conditions. Historical and current estimates of ponderosa pine forest (a) basal areas, (b) stem densities, and (c) canopy cover in or near Salt River watershed. Lines bisecting boxes represent median values; lower and upper box borders represent first and third quartile values; whiskers extend 1.5 interquartile ranges below and above boxes. Outlier points are not displayed for visual clarity. Historical basal area and stem density from sites in the Southwest, representing conditions from 1867–1925 [86]. Historical canopy cover from sites in northern Arizona adjacent to study area, representing conditions from 1873–1874 [85]. 1950s basal area from sites in the Salt River watershed from 1951–1952 [71]. Current conditions from US Forest Inventory and Analysis (FIA) program plots [87,88] and the Integrated Landscape Assessment Project Model (ILAP) extracted within in the Salt River watershed boundary [89].

Based on measurements from multiple forest inventories collected in or near the Salt watershed [85–89], basal areas of ponderosa pine forests doubled, canopy cover increased 2–3 fold, and tree densities increased at least 10-fold from a historical period (1867–1925) to a current period (2000–2012) (Fig 4). Though the sample size was small, a forest study conducted in the watershed from 1951–1952 [71] suggested that the majority of these changes occurred in the first half of the century. Basal areas in these forest stands were near the median of current forest stands and above the range of historical conditions (Fig 4a).

**Fig 5:**
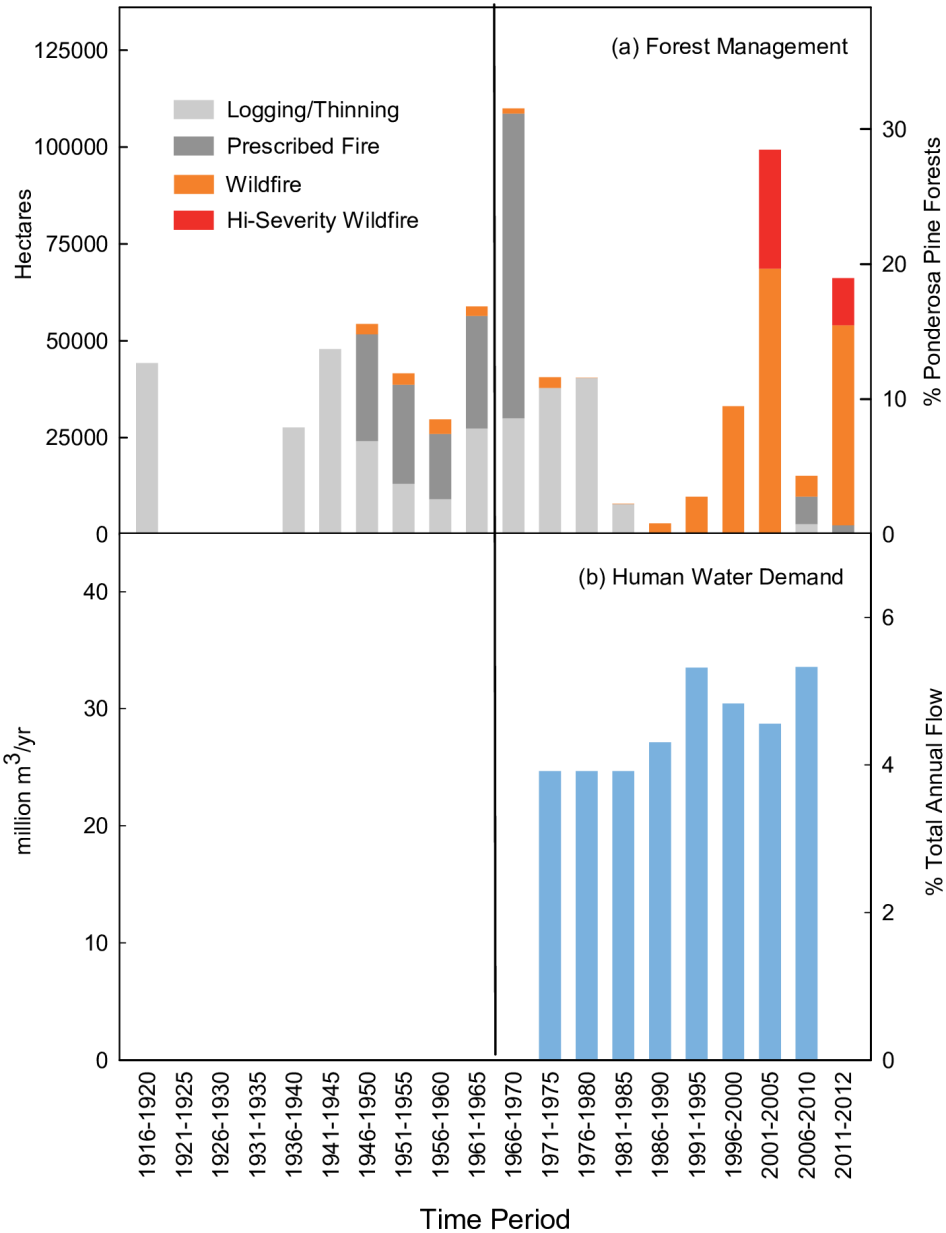
Estimates of historical forest management and water demand. Qualitative summaries of 20^th^ century (a) forest management activities and (b) human water demand in Salt watershed in central Arizona in 5-year increments. Activities in (a) are shown as watershed areas affected by logging/thinning [90], prescribed fires [90,91] and wildfires [90–92] in hectares and as percentage of ponderosa pine forests in the study watershed. Annual groundwater use, surface water use and surface water diversions shown in million m^3^/year and as percentage of mean annual flows of Salt River near Roosevelt [50,93]. Vertical line in middle of panels denotes rough break between 1914–1963 and 1964–2012 study time periods. See Table 3 and methods for detailed description of available data and data gaps.

These changes in forest conditions due to a century of fire suppression were likely representative of the majority of ponderosa pine forests in the watershed because forest management activities generally occurred on less than 30% of the forests in the watershed (Fig 5a). While there were several data gaps in the forest management and wildfire history in the watershed (Table 3), historical accounts in the region suggested it was unlikely that the pace of logging exceeded levels where data were available [90,100]. Nor were there any records of large wildfires that occurred in the watershed prior to 1970.

The fire suppression policy was effective until recent decades such that areas burned in wildfires in any five-year interval before the 1990s ranged from 0–2,900 hectares (0–7,200 acres) (Fig 5a). Thereafter, wildfire activity increased dramatically when the two largest wildfires in Arizona state history, the Rodeo-Chediski fire in 2002 and the Wallow fire in 2011, burned 28% and 18% of the watershed’s ponderosa pine forests, respectively. A significant portion of these wildfires burned under high-severity conditions.

### Changes in human water use

Total human water demand in the watershed was low, near or below 5% of the mean annual flow at the Salt River near Roosevelt streamgage from 1970–2010 (Fig 5b) [50,93]. Though data from previous years were not available, it is unlikely that pre-1970 water use was substantially greater than recent decades given the sparse population and remote location of the watershed.

### Climate trends

**Fig 6:**
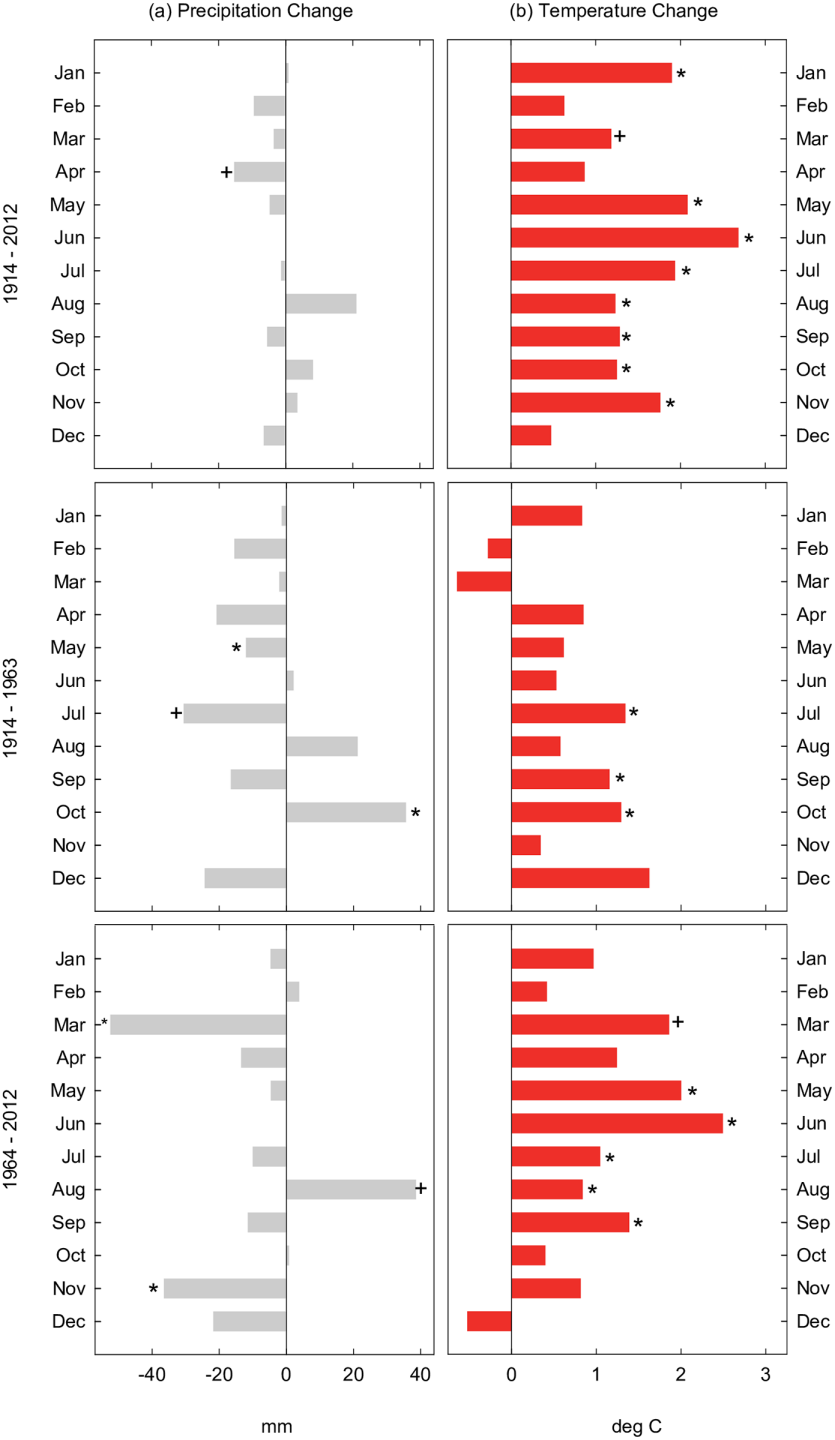
Changes in precipitation and temperature. Estimated magnitudes of change in (a) monthly precipitation (mm) and (b) monthly temperature (°C) across three study periods in Salt watershed, Arizona based on data from McNary weather station near Pinetop, Arizona (NWS Station #025412) [61]. Monthly changes derived from Sen’s slope estimate multiplied by number of years in period. Significance levels (* p-value < = 0.05, + p-value < = 0.1) based on Mann-Kendall test.

Monthly precipitation was highly variable in the Salt River watershed in the last century (Fig 6a). A few months had significant trends over the periods evaluated but overall there was no discernable pattern of precipitation change across seasons or years. Trends in monthly snowpack levels from 1964–2012 were also not consistent, though the Regional Kendall test showed that February levels declined at near significant level (Table 4). In contrast, temperature change was monotonic across the century, with significant increases of 1–3°C in 8 of the 12 months across the full study period (Fig 6b). Temperature change was of greater magnitude and significant in more months in the second sub-period than the first.

**Table 4:** Trends in snowpack, 1964–2012.

		Site Mann-Kendall Trend test			
		1 Feb SWE		1 Mar SWE	
Site	Elevation (m)	Slope	p-value	Slope	p-value
Chalender	2164	-0.458	0.281	-0.429	0.457
Fort Valley	2240	0.000	0.762	0.000	0.768
Happy Jack	2326	0.000	0.775	0.000	0.749
Beaver Head	2436	-0.363	0.398	-0.175	0.569
Coronado Trail	2545	0.000	0.543	-0.391	0.136
Nutrioso	2591	-0.492	0.113	-0.455	***0*.*074***
Fort Apache	2792	0.224	0.769	0.000	1.000
		Regional Kendall Trend test			
Salt Watershed	-	-0.224	***0*.*095***	-0.143	0.141

Trends in 1 Feb SWE and 1 Mar SWE snowpack levels in or near Salt River watershed, Arizona from 1964–2012. Slope estimates based on Sen’s method. Trend significance level based on Mann-Kendall test for individual sites and the Regional Kendall test for the Salt watershed (values in bold-italics were nearly significant at p-value < = 0.1).

#### Flow trends

**Fig 7:**
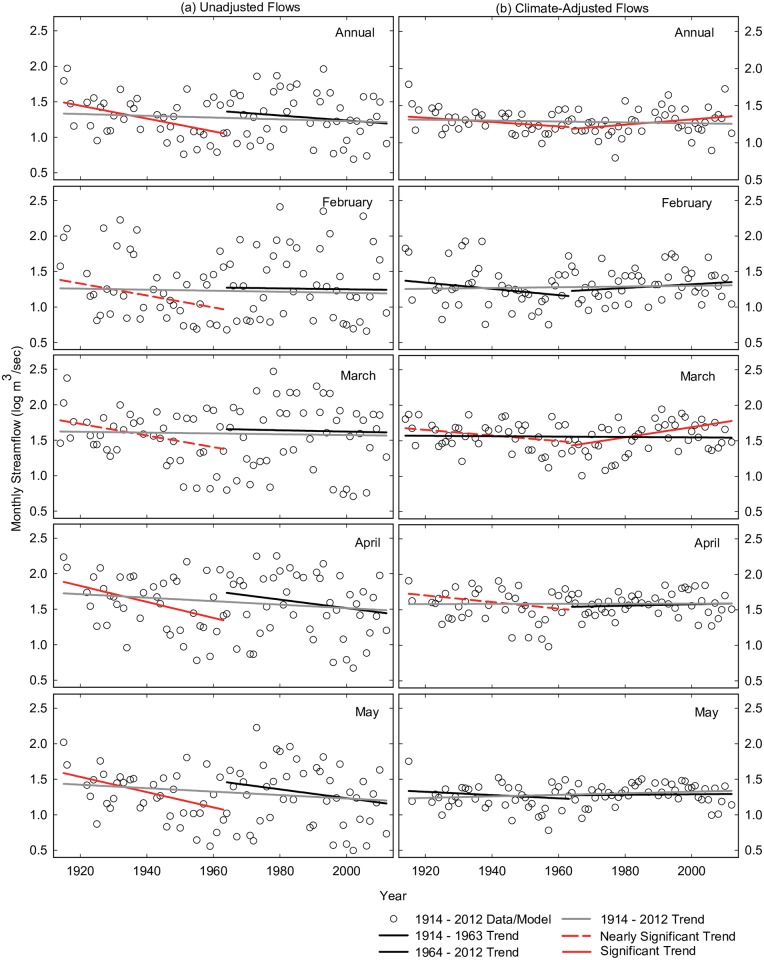
Trends in annual, February–May streamflow. Time series and trends in (a) annual/monthly flows and (b) annual/monthly flows adjusted for variation in precipitation and temperature in the Salt River near Roosevelt, Arizona. February to May monthly flow estimates are shown. Vertical axes are units are log m^3^ s^-1^. Dots in (a) are unadjusted flow values and (b) are residuals (+ mean) from regression model built from full dataset (1914–2012). Lines represent trends across three study periods using Sen’s estimate of slope for (a) data and (b) model residuals (+ mean) from each corresponding time-period. Trend significance based on Mann-Kendall test. See Table 5 and methods for detailed information on regression models used to derive climate-adjusted flows and trends.

**Fig 8:**
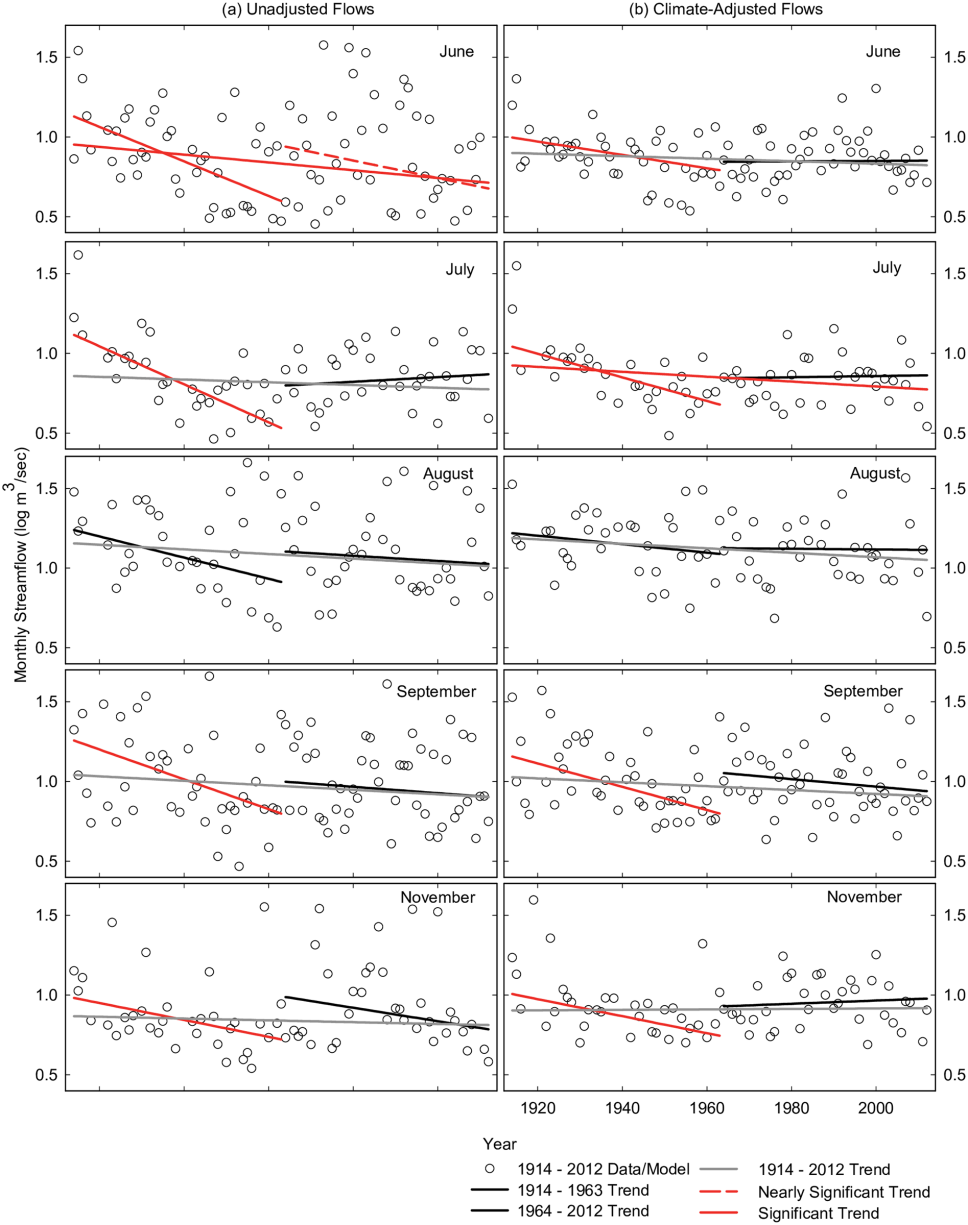
Trends in monthly streamflow, June–September, November. As in Fig 7, but for monthly flows (log m^3^ s^-1^) in June–September and November. Data and trends for July, August, and November represent 75% of available data to remove serial correlation of model residuals.

**Fig 9:**
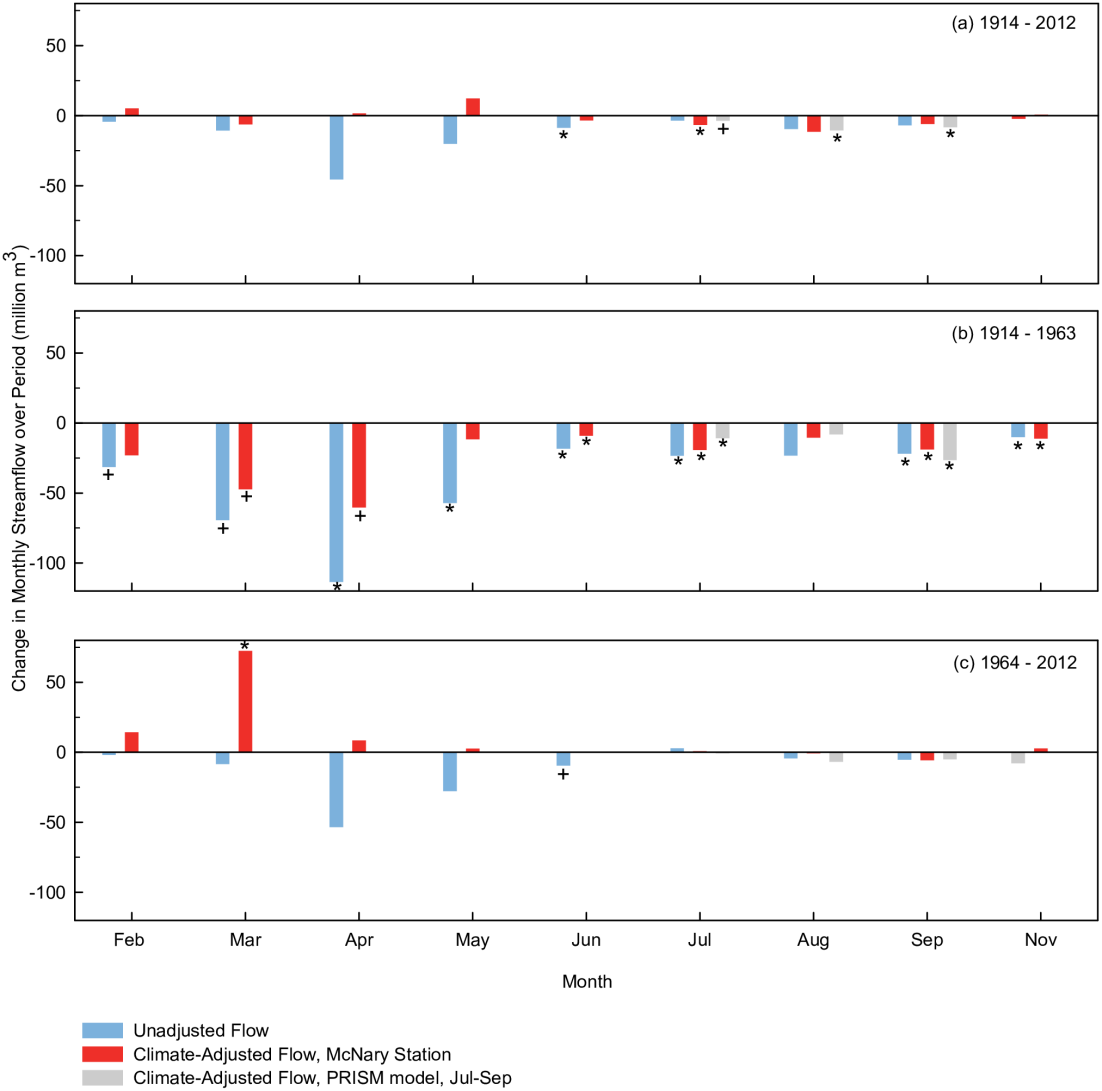
Changes in monthly streamflow. Monthly trends from Figs 7 and 8, shown as changes of magnitude in streamflow (million m^3^) in Salt watershed near Roosevelt, Arizona from (a) 1914–2012, (b) 1914–1963, and (c) 1964–2012. Bars show magnitude estimates for unadjusted flows (blue), flows adjusted for precipitation and temperature using McNary weather station (red) and PRISM model (grey). Monthly changes derived from Sen’s slope estimate multiplied by number of years in period and converted to volumes. Significance levels (* p-value < = 0.05, + p-value < = 0.1) based on Mann-Kendall test.

Annual and monthly flows did not significantly change across the century except for a decline in June flows (Figs 7a and 8a). In contrast, flows from 1914–1963 declined significantly at the annual time step and in six months (April, May, June, July, September, November) and declined at near significant levels in two spring months (February, March). There were no significant flow trends from 1964–2012 (near significant decline in June). In terms of magnitudes, changes in flow were largest in the 1914–1963 time-period and largest in spring months (Fig 9b, blue bars). The range of annual flow declines in this period was 245 million m^3^ (198,000 acre-feet) to 521 million m^3^ (422,000 acre-feet), a 32–69% decline, based upon significant trends at the monthly and annual time steps respectively. As a percentage of mean flows, monthly flows declined by 45–81% in those months with significant or near significant trends. Because we used Sen’s method to estimate trend slopes [96], these magnitudes can be thought of as changes in median flow from the beginning of the period to the end of the period.

#### Climate-flow regression models

Linear regression models indicated that precipitation was a consistent predictor of flow that explained the most variability across all the time periods evaluated, whereas the influence of temperature varied by season and time-period (Table 5, S1 Table). Temperature was most consistent as a predictor of flow across the full period (significant in 8 of 9 months evaluated), somewhat consistent from 1964–2012 (annual, 6 of 9 months), and less consistent from 1914–1963 (4 of 9 months). It was a more consistent predictor of flow variability in spring months than in monsoon-influenced summer months. The slope coefficients in the regression models were positive for precipitation and negative, in most cases, for temperature indicating lower flows were associated with lower precipitation and higher temperatures, respectively. Regression models for snow-influenced winter and spring months used quadratic precipitation terms to improve model fit, whereas linear precipitation terms were selected for regression models in summer months and at the annual time step. We were not able to develop monthly flow models for any time-period in October, December, and January because the distribution of errors in the residual plots was not normally distributed.

**Table 5:** Climate-flow regression models.

Model						Precipitation						Temperature					
Monthly Flow	Model Type	Time Period	# Years	adj r^2^	SE	Prec 1	Sign	p-value	Prec 2	Sign	p-value	Temp 1	Sign	p-value	Temp 2	Sign	p-value
Annual	Linear P	1915–2012	84	0.709	0.169	Jul-Jun	+	***									
"	Linear P	1915–1963	39	0.721	0.152	Jul-Jun	+	***									
"	Linear P, T	1964–2012	45	0.728	0.177	Jul-Jun	+	***				Jul-Jun	-	*			
February	Quadratic P, T	1914–2012	88	0.668	0.270	Dec-Feb	+	***	(Dec-Feb)^2^	-	*	Nov	-	*	Feb	+	***
"	Quadratic P, T	1914–1963	42	0.497	0.311	Dec-Feb	+	***	(Dec-Feb)^2^	-	*				Feb	+	**
"	Quadratic P, T	1964–2012	46	0.812	0.216	Dec-Feb	+	***	(Dec-Feb)^2^	-	N.S.	Nov	-	***	Feb	+	*
March	Quadratic P, T	1914–2012	88	0.751	0.212	Nov-Mar	+	***	(Nov-Mar)^2^	-	***	Mar	+	*			
"	Quadratic P	1914–1963	42	0.637	0.205	Nov-Mar	+	***	(Nov-Mar)^2^	-	*						
"	Quadratic P	1964–2012	46	0.778	0.232	Nov-Mar	+	***	(Nov-Mar)^2^	-	***						
April	Quadratic P, T	1914–2012	86	0.769	0.199	Dec-Apr	+	***	(Dec-Apr)^2^	-	**	Nov-Jan	-	***	Mar	-	***
"	Quadratic P, T	1914–1963	40	0.595	0.247	Dec-Apr	+	**	(Dec-Apr)^2^	-	N.S.				Mar	-	***
"	Quadratic P, T	1964–2012	46	0.879	0.153	Dec-Apr	+	***	(Dec-Apr)^2^	-	***	Nov-Jan	-	***	Mar	-	*
May	Quadratic P, T	1914–2012	85	0.820	0.167	Dec-May	+	***	(Dec-May)^2^	-	**	Nov-Dec	-	*	Mar-Apr	-	***
"	Quadratic P, T	1914–1963	39	0.657	0.198	Dec-May	+	***	(Dec-May)^2^	-	*				Mar-Apr	-	***
"	Quadratic P, T	1964–2012	46	0.896	0.141	Dec-May	+	***	(Dec-May)^2^	-	**	Nov-Dec	-	*	Mar-Apr	-	***
June	Quadratic P, T	1914–2012	89	0.732	0.157	Nov-May	+	***	(Nov-May)^2^	-	N.S.	Apr-May	-	***			
"	Quadratic P, T	1914–1963	43	0.604	0.171	Nov-May	+	*	(Nov-May)^2^	-	N.S.	Apr-May	-	*			
"	Quadratic P, T	1964–2012	46	0.801	0.149	Nov-May	+	***	(Nov-May)^2^	-	N.S.	Apr-May	-	*			
July75	Linear P	1914–2012	67	0.278	0.181	Jan-Mar	+	**	Jul	+	***						
"	Linear P	1914–1963	33	0.279	0.214				Jul	+	***						
"	Linear P	1964–2012	34	0.280	0.144	Jan-Mar	+	*	Jul	+	**						
August75	Linear P, T	1914–2012	69	0.405	0.210	Jul-Aug	+	***				Aug	-	**			
"	Linear P	1914–1963	33	0.523	0.186	Jul-Aug	+	***									
"	Linear P, T	1964–2012	36	0.369	0.221	Jul-Aug	+	***				Aug	-	**			
September	Linear P, T	1914–2012	92	0.435	0.211	Aug	+	***	Sep	+	***	Sep	-	**			
"	Linear P	1914–1963	44	0.475	0.230	Aug	+	**	Sep	+	***						
"	Linear P, T	1964–2012	48	0.382	0.193	Aug	+	*	Sep	+	***	Sep	-	*			
November75	Linear P, T	1914–2012	71	0.552	0.190	Oct	+	***	Nov	+	***	Oct	-	*			
"	Linear P	1914–1963	35	0.205	0.242	Oct	+	*	Nov	+	**						
"	Linear P	1964–2012	36	0.809	0.130	Oct	+	***	Nov	+	***						

Overall, the models explained the most variation of flow at the annual time step (mean adjusted r^2^ = 0.72, range: 0.71–0.73) and in spring months (mean adjusted r^2^ = 0.73, range: 0.50–0.90) and the lowest amount of variation in summer months (mean adjusted r^2^ = 0.38, range: 0.28–0.55). When temperature terms were significant, they explained, on average, an additional 7% of flow variation than was explained by precipitation-only models (S1 Table). Models that used the more spatially extensive PRISM model for precipitation and temperature predictor variables explained more variation in summer flow (July-September, mean adjusted r^2^ = 0.62, range: 0.50–0.81) than models using McNary weather station data (S1 Table).

Multiple linear regression models explaining variation in annual and monthly total streamflow for the Salt River near Roosevelt, Arizona [60] based on precipitation and temperature at McNary, Arizona [61]. Models built with full dataset (1914–2012) and re-run for two sub-periods. Final models included explanatory variables that were significant (* < = 0.05, ** < = 0.01, *** < = 0.001) and not correlated, except for precipitation polynomial terms that were needed to satisfy assumptions of linear fit (N.S. denotes non-significance for these terms). Months labeled with ‘75’ indicate only 75% of data were used to eliminate serial correlation of model residuals. Regression models described in more detail in methods section and S1 Table.

#### Climate-adjusted flow trends

After adjusting for precipitation and temperature, annual flows did not change significantly across the century, but did decrease significantly from 1914–1963 and increase significantly from 1964–2012 (Fig 7b), suggesting different processes were altering the water budget in these two periods. At the monthly time step, July was the only month in which flows adjusted for precipitation and temperature declined significantly across the full century (Figs 7b and 8b). Models that used the PRISM model for predictor variables to explain summer flows produced trends that were of similar magnitudes and significance levels to McNary station regression models (Fig 9, grey and bars).

From 1914–1963, monthly adjusted flows declined significantly in most summer and fall months (June, July, September, November), and declined at near significant levels in two spring months (March, April). The range of annual flow declines adjusted for precipitation and temperature in this period was 59 million m^3^ (47,500 acre-feet) to 216 million m^3^ (175,000 acre-feet) based on significant trends drawn from the monthly (Fig 9b, red bars) and annual time steps respectively. Reductions amounted to an 8–29% decline in annual flows (compared to the period mean) and a 37–56% decline in summer and fall months.

Contrary to the study hypotheses, flows did not decline from 1964–2012 after accounting for precipitation and temperature or precipitation-alone (Table 6). Rather, adjusted flows increased significantly in March and at the annual time step (Fig 7b). Climate-adjusted increases in annual flow were 73 million m^3^ (58,800 acre-feet) to 222 million m^3^ (180,000 acre-feet) based on significant trends at the monthly (Fig 9c, red bars) and annual time steps respectively, a 10–31% gain (compared to period mean).

**Table 6:** Trends in streamflow adjusted for precipitation, 1964–2012.

Monthly Flow	Trend Significance	Change in Streamflow (million m^3^)	% Change
February	N.S.	6.26	6%
**March**	*	**72.51**	**45%**
April	N.S.	-2.06	-2%
May	N.S.	-5.19	-7%
June	N.S.	-1.45	-6%
July75	N.S.	0.75	4%
August75	N.S.	-12.45	-31%
**September**	**+**	**-9.17**	**-31%**
November75	N.S.	2.75	10%

Estimated magnitudes of change (million m^3^) and percent change from mean in monthly streamflow adjusted for variation in precipitation in Salt watershed near Roosevelt, Arizona from 1964–2012. Monthly changes derived from Sen’s slope estimate multiplied by number of years in period and converted to volumes. Significant or nearly significant trends in bold (* p-value < = 0.05, + p-value < = 0.1), based on Mann-Kendall test. ‘N.S.’ indicates trend was not significant. Precipitation explanatory variables were the same as those indicated in Table 5. Months labeled with ‘75’ indicate only 75% of data were used to eliminate serial correlation of flow residuals.

### Changes in flow timing

**Fig 10:**
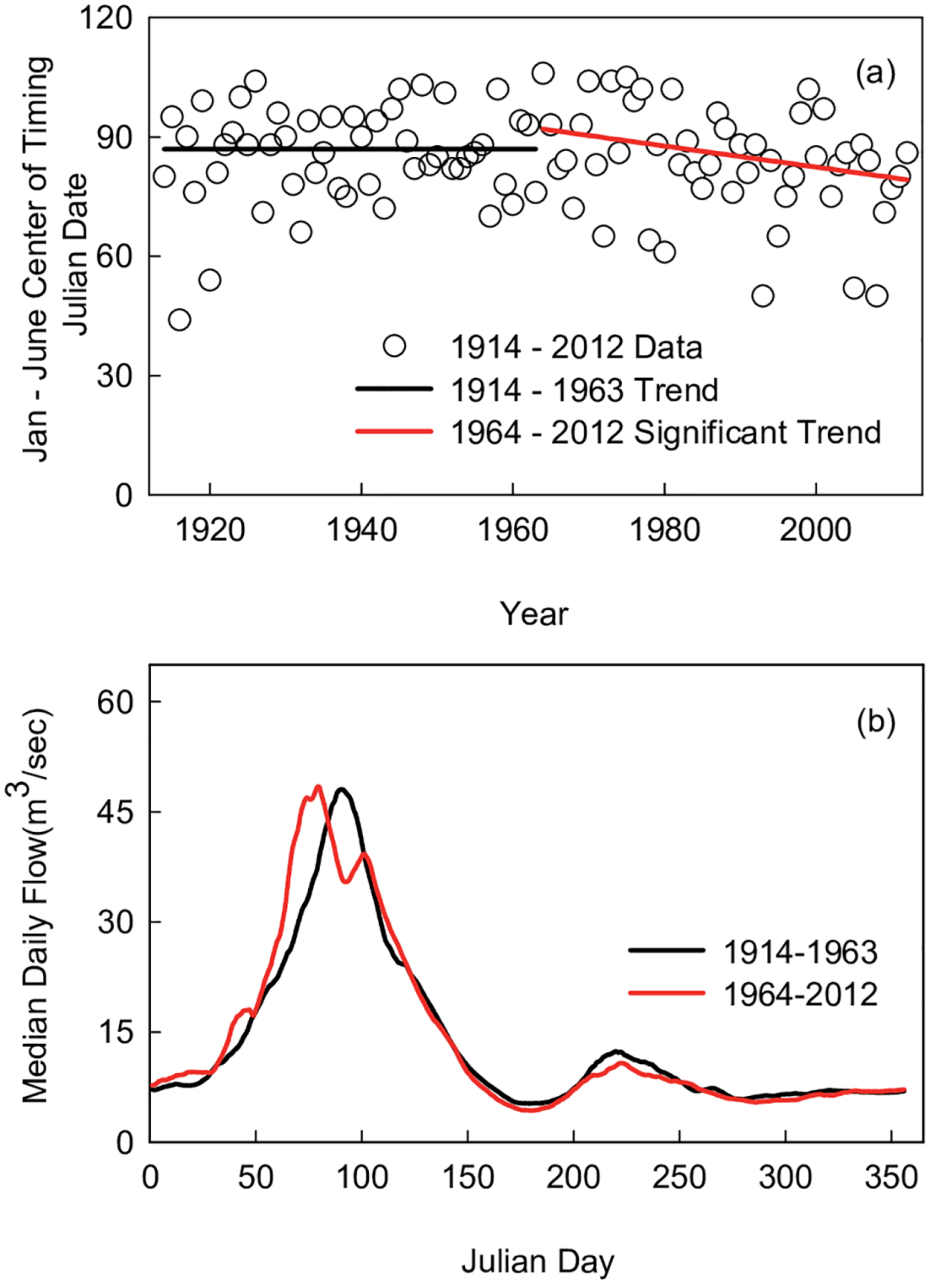
Changes in timing of peak spring flow. Changes in timing of peak streamflow during spring months in Salt River near Roosevelt, Arizona, across two study periods. Lines in (a) represent Sen’s slope estimate of trends for date at which 50% of flows from January–June was reached. Significance level based on Mann-Kendall test. Lines in (b) represent median daily flows smoothed by 10-day average in 1914–1963 (black) and 1964–2012 (red).

Peak spring flows shifted towards earlier dates in March from 1964–2012. The trend of the date at which 50% of January-June flow was reached advanced significantly in this period by 12 days, from a median of April 2 at beginning of the period to median of March 21 at the end of the period (Fig 10a). There was no trend in this date from 1914–1963. Using a 10-day rolling average of median daily flow, Fig 10b shows visually how peak flows in the second sub-period occurred earlier than peak flows in the first sub-period. In the regression models, February and March flows were associated with positive coefficients for temperature terms in the same month (Table 5, S1 Table), indicating that warmer temperatures increased spring flows in these months.

## Discussion

We evaluated 20^th^ century trends in streamflow, climate and forest conditions in the Salt River watershed in central Arizona and found that precipitation trends were variable across the century, temperatures rose, forest densities increased, and streamflow declined most consistently in the 1^st^ half of the century. Linear regression models showed that precipitation was the dominant factor that explained variation in flows. After accounting for climate variation, the only factor that we examined that could account for an 8–29% decline in annual streamflow from 1914–1963 was a 2-fold increase in forest basal area, a 2-3-fold increase in canopy cover, and at least a 10-fold increase in forest density (Fig 4) associated with fire suppression. In the second half of the century, warmer temperatures were associated with shift toward earlier timing of peak spring flows. Rather than the anticipated decline in seasonal flows associated with changes in forest and temperature conditions, we found that climate-adjusted flows from 1964–2012 increased in March and at the annual time step. At least a portion of enhanced March flows was likely due to a 12-day shift towards earlier peak spring flows in late March. Thus, we conclude that directional changes in forest and temperature conditions influenced 20^th^ century flow in the Salt watershed but that their signature of influence varied by time-period and season.

### Forest influence

Two other regional studies have found increases in forest densities that were associated with flow declines. In a municipal watershed for Santa Fe, New Mexico, a forest restoration and protection program was put into place after early 20^th^ century logging activities. As forest densities increased under this program, annual flows in that watershed declined by approximately 25% from 1913–1957 after accounting for precipitation (decline significant at p-value < 0.1) [53]. Using a simulation model, Covington and Moore [44] estimated that streamflow in the Verde River, adjacent to our study area, declined by 26% between 1867 and 1987 due to increases in forest density and basal area. Other studies in the western US have found declines in summer flows [14,16,52,77], although these studies did not account for forest conditions and the majority of the streamflow changes occurred post mid-century with warmer temperatures. By separating climate from non-climate effects, this study adds to the knowledge gained in other regions that forest change can modulate climate variability effects on flow [33–36], and in this case, well before the influence of anthropogenic warming.

We anticipated denser forests would depress streamflow throughout the 20^th^ century, but found no evidence for this hypothesis in the 2^nd^ half of the century. A potential explanation for this discrepancy, supported by several forest studies conducted in central Arizona, is that forest change primarily occurred in the 1^st^ half of the century and slowed down at some point in the middle of the century where perhaps forest conditions reached a new equilibrium due to water limitations. A large ponderosa pine recruitment event occurred in central Arizona in 1919 [101] and in subsequent decades, tree densities increased with fire suppression, especially within small-diameter age classes [70]. By mid-century, forest stands in or near to the Salt watershed already had very high basal areas [71], and these stands exhibited very low growth rates [70,71,102,103]. An experimental study in the neighboring Verde River watershed found that forest thinning commenced at mid-century increased tree growth rates in thinned stands while growth in un-thinned control stands continued at low rates [102]. Another study found that increases in density and basal areas peaked in the 1970s and declined thereafter in association with higher rates of tree mortality [70]. Water availability is known to be the greatest limiting factor for tree growth and densities within ponderosa pine forests in the arid Southwest [73,74,104,105].

### Warming influence

Warmer winter and spring temperatures were associated with earlier timing of peak spring flows in the 2^nd^ half of the century, presumably due to more sensible and latent heat available for snowmelt. Ellis and Sauter [99] and Clow [10] used regression models to determine that warmer spring temperatures were a significant predictor of earlier spring flows in the Salt River and Colorado watersheds, respectively. The 12-day shift towards earlier peak flows that began at mid-century was well within the 1–4 week advance in timing reported for other basins in the western United States [11], and the increase in climate-adjusted March flows was of similar magnitude to other regional studies [106,107]. It is likely that at least a portion of climate-adjusted flow increases in March (Fig 7b) was due to an increase in runoff within the month from earlier timing of snowmelt.

Beyond a shift in spring timing, the fact that annual flows increased after climate-adjustment from 1964–2012 (Fig 7b) suggests that other changes to the water budget occurred in this period. An alternative hypothesis for this finding was that warming resulted in more winter rainfall and a greater proportion of runoff generated from rainfall in this period. An increase in winter rainfall and a decrease in snowfall would be expected to reduce evapotranspiration losses and increase runoff efficiencies because a greater proportion of moisture would effectively bypass snowpack sublimation, peak growing season transpiration, and peak summer evaporation. Other regional studies have detected higher winter and spring rainfall at mid- to low-elevations in recent decades [7,9,14,108]. Svoma [78] found that the elevation above which snow accumulates in the Salt watershed (e.g. snowline) has risen by an approximately 200 meters (~2,200 m) in the last several decades [78]. This suggests that under sufficient moisture conditions approximately 75% of the watershed (10% more of than previous period) could have generated runoff from rainfall. While their linkage to anthropogenic warming is unclear, extreme winter rainfall events and subsequent flooding have occurred in the last several decades in Arizona [48,84]. Atmospheric rivers can increase the proportion of runoff caused by rainfall because they tend to occur under warmer winter temperatures. In the Salt river watershed from 1979–2009, they accounted for higher soil moisture levels and runoff coefficients than under normal conditions [83].

### Study limitations

We employed statistical approaches to evaluate associations between streamflow, forest conditions, and temperature changes. Uncertainties in our statistical approach included limited spatial and temporal data for climate and especially forest conditions, inaccuracy in regression model parameters, model error, and lack of examination of interactions between temperature, precipitation, and snowpack. Our linear regression models were relatively simple and only accounted for seasonal precipitation totals and mean temperature conditions. As such, the models may not have captured complex and perhaps non-linear warming effects on intermediate storage pools (processes), such as snowpacks (sublimation) and soil moisture (evapotranspiration), changes in rainfall versus snowpack-generated runoff, or the influence of extreme precipitation events (< 1 month) [48,83,84].

Additionally, monsoon rainstorms are known to be spatially variable such that data from one weather station may not have fully captured this variability. The performance of the linear regression models in explaining flow variation in summer months was relatively low compared to models developed for other seasons (Table 5). This limitation was somewhat lessened with models that used the more spatially extensive PRISM model where precipitation and temperature explanatory terms were estimated across the entire watershed surface [66]. These models explained a higher percentage of summer flow variability and the residual flow trends showed significant declines in the same months and had similar magnitude to residuals from weather station models (Fig 9).

It is possible that losses in seasonal flows were stabilized or reversed due to the heightened wildfire activity within the watershed that began in the 1990s. A recent study in forested watersheds in New Mexico found that runoff increased in two of three large watersheds when at least one-fifth of these watersheds had burned in the previous 3–5 years [109]. Areas burned in the Salt watershed approached or exceeded these levels in 2^nd^ half of the 20^th^ century (Fig 5), and these estimates are likely conservative because prescribed fire data was unavailable from tribal and US Forest Service lands for much of this time-period (Table 3). Wildfires reduce evapotranspiration losses [31] in comparison to high-density forests and could also generate intermediate canopy densities that are conducive to high snowpack levels [32,55], although field studies at hillslope to watershed spatial scales have found that severe forest disturbances can reduce snowpack and streamflow by increasing sublimation and evaporation losses [110–112]. A better understanding of the hydrological effects of forest disturbances is urgently need because their extent and severity are projected to increase in the future, potentially resulting in broad-scale tree mortality and deforestation [113–115].

### Research and management implications

Southwestern US watersheds have experienced substantial changes in ponderosa pine forest cover due to fire suppression [42–47] and may be particularly sensitive to warming effects on snowpack and rain/snow ratios [108], but the influence of these factors is obscured by the dominant influence of precipitation [28,29,39]. We recommend that future flow trend analyses in this region systematically account for variability in precipitation, which is fluctuating between dry and wet cycles [13,19,20], so that more information could be brought to bear on factors that are experiencing directional change. Analyses and models that evaluate fluctuations of rain and snow with temperatures hold some promise of disentangling how warming changes precipitation phases and subsequent flows [99,108]. Process-based hydrological models that are calibrated with local watershed, climate, and land use parameters can be used to monitor spatial and temporal changes in snowpack and streamflow [116]. Remote sensing technologies can be leveraged to establish snowpack and forest cover relations at larger scales [32], and at fine scales, drones can be used to compare soil moisture and snowpack levels before and after forest thinning treatments. Potentially more costly measures can be taken in watersheds that provide water supply to large population centers, such as paired watershed experiments that use modern instrumentation like flux towers to provide more refined information about forest management impacts on hydrology [117].

Concerted efforts in research and monitoring are needed given the critical role that mountain forests play for people and nature. Sustained or continued flow declines in summer months may affect water providers who often face shortages during this period, and these flows are critical to the survival of water-dependent natural resources. Declines in fish and riparian species diversity [118–120] and increases in riparian tree stress and mortality [121,122] have been associated with low or intermittent seasonal flows, low groundwater levels, and drought conditions in the Southwest. Modeling suggests that flows and groundwater recharge in central Arizona will decline in the future, due to climate change [21,123–125] and rising human water demand [126], further imperiling water-dependent species.

Land and water managers will likely have fewer options available to sustain natural resources and meet objectives in the future. A critical evaluation of the three factors that control flow in southwestern US rivers—drought, warmer temperatures, and forest conditions—indicates that managers can only directly and reasonably influence forest conditions. Thinned forests have slightly lower evapotranspiration losses than un-thinned forests [31], a finding that cannot be discounted in a region with scarce water resources where roughly 90% of incoming precipitation is lost to evapotranspiration. To put this in perspective, if basal areas were reduced from current conditions to historical conditions (22 to 12 m^2^/ha), annual flows in the Salt River watershed would increase by 14% (112 million m^3^, 91,000 acre-feet) according to a model adapted from historical forest watershed experiments [98].

These studies and others suggest that regional forest restoration projects that increase the scale and pace of forest restoration activities, such as the Four Forest Restoration Initiative [127], hold some promise of recovering seasonal flows that were presumably lost due to rapid forest densification in the first half of the 20^th^ century. Any potential gains in flow associated with forest thinning would likely to be short-lived due to understory regrowth if initial treatments are not maintained [28,39], though it is possible that prescribed fires that will follow initial thinning treatments [127], or allowing wildfires to burn under appropriate conditions could sustain at least a portion of these gains [109]. Therefore, the most practical course of action may be to accelerate the pace and scale of forest restoration programs designed to reduce wildfire risks, safeguard cities, and improve forest resilience in the short term. Moving forward, researchers can continue to translate knowledge from forest hydrology research and monitoring efforts to managers with better tools to evaluate the co-benefits and trade-offs that restoration practices have on ecosystem resilience and services, including water provision and carbon sequestration [24].

## Supporting information

S1 Table Flow regression models. Information on all regression models developed in study including model adjusted r^2^ and standard error and predictor variables coefficients and significance levels.
(XLSX)

S1 Text Extension and analysis of weather station records. (DOCX)
