# Correction: A century of changing flows: Forest management changed flow magnitudes and warming advanced the timing of flow in a southwestern US river

**DOI:** 10.1371/journal.pone.0191443

**Published:** 2018-01-12

**Authors:** Marcos D. Robles, Dale S. Turner, Jeanmarie A. Haney

The legend for Table 5 is incorrect. The text of the legend is incorrectly placed within the main article text as the third paragraph of the “Climate-flow regression models” heading of the Climate trends subsection of the Results. This paragraph should instead be included as the legend for Table 5. Please see the correct table and legend here.

**Table 5:** Climate-flow regression models. Multiple linear regression models explaining variation in annual and monthly total streamflow for the Salt River near Roosevelt, Arizona [60] based on precipitation and temperature at McNary, Arizona [61]. Models built with full dataset (1914–2012) and re-run for two sub-periods. Final models included explanatory variables that were significant (* < = 0.05, ** < = 0.01, *** < = 0.001) and not correlated, except for precipitation polynomial terms that were needed to satisfy assumptions of linear fit (N.S. denotes non-significance for these terms). Months labeled with ‘75’ indicate only 75% of data were used to eliminate serial correlation of model residuals. Regression models described in more detail in methods section and S1 Table.

Model						Precipitation						Temperature					
Monthly Flow	Model Type	Time Period	# Years	adj r^2^	SE	Prec 1	Sign	p-value	Prec 2	Sign	p-value	Temp 1	Sign	p-value	Temp 2	Sign	p-value
Annual	Linear P	1915–2012	84	0.709	0.169	Jul-Jun	+	***									
"	Linear P	1915–1963	39	0.721	0.152	Jul-Jun	+	***									
"	Linear P, T	1964–2012	45	0.728	0.177	Jul-Jun	+	***				Jul-Jun	-	*			
February	Quadratic P, T	1914–2012	88	0.668	0.270	Dec-Feb	+	***	(Dec-Feb)^2^	-	*	Nov	-	*	Feb	+	***
"	Quadratic P, T	1914–1963	42	0.497	0.311	Dec-Feb	+	***	(Dec-Feb)^2^	-	*				Feb	+	**
"	Quadratic P, T	1964–2012	46	0.812	0.216	Dec-Feb	+	***	(Dec-Feb)^2^	-	N.S.	Nov	-	***	Feb	+	*
March	Quadratic P, T	1914–2012	88	0.751	0.212	Nov-Mar	+	***	(Nov-Mar)^2^	-	***	Mar	+	*			
"	Quadratic P	1914–1963	42	0.637	0.205	Nov-Mar	+	***	(Nov-Mar)^2^	-	*						
"	Quadratic P	1964–2012	46	0.778	0.232	Nov-Mar	+	***	(Nov-Mar)^2^	-	***						
April	Quadratic P, T	1914–2012	86	0.769	0.199	Dec-Apr	+	***	(Dec-Apr)^2^	-	**	Nov-Jan	-	***	Mar	-	***
"	Quadratic P, T	1914–1963	40	0.595	0.247	Dec-Apr	+	**	(Dec-Apr)^2^	-	N.S.				Mar	-	***
"	Quadratic P, T	1964–2012	46	0.879	0.153	Dec-Apr	+	***	(Dec-Apr)^2^	-	***	Nov-Jan	-	***	Mar	-	*
May	Quadratic P, T	1914–2012	85	0.820	0.167	Dec-May	+	***	(Dec-May)^2^	-	**	Nov-Dec	-	*	Mar-Apr	-	***
"	Quadratic P, T	1914–1963	39	0.657	0.198	Dec-May	+	***	(Dec-May)^2^	-	*				Mar-Apr	-	***
"	Quadratic P, T	1964–2012	46	0.896	0.141	Dec-May	+	***	(Dec-May)^2^	-	**	Nov-Dec	-	*	Mar-Apr	-	***
June	Quadratic P, T	1914–2012	89	0.732	0.157	Nov-May	+	***	(Nov-May)^2^	-	N.S.	Apr-May	-	***			
"	Quadratic P, T	1914–1963	43	0.604	0.171	Nov-May	+	*	(Nov-May)^2^	-	N.S.	Apr-May	-	*			
"	Quadratic P, T	1964–2012	46	0.801	0.149	Nov-May	+	***	(Nov-May)^2^	-	N.S.	Apr-May	-	*			
July75	Linear P	1914–2012	67	0.278	0.181	Jan-Mar	+	**	Jul	+	***						
"	Linear P	1914–1963	33	0.279	0.214				Jul	+	***						
"	Linear P	1964–2012	34	0.280	0.144	Jan-Mar	+	*	Jul	+	**						
August75	Linear P, T	1914–2012	69	0.405	0.210	Jul-Aug	+	***				Aug	-	**			
"	Linear P	1914–1963	33	0.523	0.186	Jul-Aug	+	***									
"	Linear P, T	1964–2012	36	0.369	0.221	Jul-Aug	+	***				Aug	-	**			
September	Linear P, T	1914–2012	92	0.435	0.211	Aug	+	***	Sep	+	***	Sep	-	**			
"	Linear P	1914–1963	44	0.475	0.230	Aug	+	**	Sep	+	***						
"	Linear P, T	1964–2012	48	0.382	0.193	Aug	+	*	Sep	+	***	Sep	-	*			
November75	Linear P, T	1914–2012	71	0.552	0.190	Oct	+	***	Nov	+	***	Oct	-	*			
"	Linear P	1914–1963	35	0.205	0.242	Oct	+	*	Nov	+	**						
"	Linear P	1964–2012	36	0.809	0.130	Oct	+	***	Nov	+	***						
