# Supplementary material for: A century of changing flows: Forest management changed flow magnitudes and warming advanced the timing of flow in a southwestern US river
Source: PLoS One. 2017 Nov 27;12(11):e0187875. doi: 10.1371/journal.pone.0187875 (PMC5703557; doi:10.1371/journal.pone.0187875)
Supplement: S1 Text — (DOCX) [file pone.0187875.s002.docx]

***S1 Text. Extension and Analysis of Precipitation Records.***

An important goal of this study was to evaluate relationships between precipitation and streamflow for the full study period 1914-2012. However, the 15 precipitation records selected were generally much shorter than the 99-year record for the Salt River (S1 Table). Only 3 sites (Alpine, Gisela, and Roosevelt 1 Wnw) had records for that time period. For detailed regression analyses, several methods were used to extend or combine some individual records into five composite records that contained data for 1914 to 2012. Roosevelt 1 Wnw had a complete record (1910-2012) and represented low elevation and low precipitation in the study watershed. Two composite records were developed to represent mid-elevations: Payson and Pleasant-Valley/Young. Two extended records represented high elevation sites: McNary and Pinetop Fish Hatchery.

Payson had a record from 1949 to 2012 and Payson Ranger Station has a record from 1910 to 1973. The two sites are about 2 miles from each other and there was a common time period of 25 years (1949-1973). To demonstrate that the two sites have similar precipitation characteristics and that it was valid to combine the records, correlation coefficients were computed for each month during the common time period. The coefficients had a minimum of 0.91, an average of 0.97, and a maximum of 0.99. Therefore, the records are highly correlated and they were combined into a composite record from 1914 to 2012. For the common time period, the average of the two monthly values from each site was used for each month in the composite record.

***S1 Table.*** ***List of Weather Stations.*** Summary of available weather station records evaluated in this study. Data from National Weather Service Cooperative Weather Stations [57]. First column identifies five composite records that were tested for detailed regression analysis with monthly flows. Suffixes in this column denote the following: ‘i’ individual record; ‘c’ composite record containing data from two individual stations; ‘e’ record of individual stations extended using other stations.

|  |  |  |  |  | Percent of |  |  |  |
| --- | --- | --- | --- | --- | --- | --- | --- | --- |
|  |  |  |  |  | possible |  | Mean |  |
|  |  |  |  |  | monthly | Land- | annual | Mean |
| Record |  |  |  | Years | obser- | surface | precipi- | annual |
| Tested in |  |  | Period of | of | vations | elevation | tation | snowfall |
| Models | ID # | Name | record | data | with data | (m) | (mm) | (mm) |
| 1 i | 27281 | Roosevelt 1 Wnw | 1910-2012 | 103 | 98 | 671 | 404 | 5 |
|  | 23448 | Gisela | 1916-2012 | 97 | 94 | 884 | 442 | 66 |
|  | 25825 | Natural Bridge | 1910-1970; 2005-2012 | 69 | 97 | 1,405 | 622 | 490 |
| 2 c | 26320 | Payson Ranger Station | 1910-1973 | 64 | 93 | 1,478 | 507 | 541 |
| 2 c | 26323 | Payson | 1949-2012 | 64 | 97 | 1,497 | 541 | 612 |
| 3 c | 26653 | Pleasant Valley Ranger Station | 1964-2012 | 49 | 96 | 1,539 | 552 | 124 |
| 3 c | 29622 | Young | 1903-1906; 1916-1964 | 53 | 90 | 1,539 | 516 | 508 |
| 4 e | 29271 | Whiteriver 1 Sw | 1910-1933; 1941-2008 | 92 | 90 | 1,561 | 479 | 427 |
|  | 20808 | Black River Pumps | 1949-2012 | 64 | 95 | 1,841 | 493 | 292 |
|  | 26581 | Pinedale | 1912-1968 | 57 | 92 | 1,984 | 475 | 1,052 |
|  | 23961 | Heber Ranger Station | 1951-2012 | 62 | 91 | 2,009 | 448 | 955 |
| 5 c | 26601 | Pinetop Fish Hatchery | 1944-2012 | 69 | 98 | 2,195 | 620 | 1,885 |
| 4 e | 25412 | McNary 2N | 1934-2012 | 79 | 97 | 2,237 | 658 | 2,250 |
|  | 20159 | Alpine | 1912-2012 | 101 | 90 | 2,454 | 507 | 1,394 |
|  | 23683 | Greer | 1957-2010 | 54 | 94 | 2,588 | 593 | 2,296 |

Pleasant Valley Ranger Station and Young are about 1 mile apart and had no common time period. To demonstrate that the two sites had similar precipitation characteristics, average monthly values of precipitation for each site were compared. The differences between each site’s average monthly values were calculated. Seven differences were positive and 5 differences were negative. The maximum negative difference was -14 percent, the average difference was 0.2 percent, and the maximum positive difference was 12 percent. The average absolute difference was 4 percent. The records were therefore sufficiently similar to combine and a composite record was created for 1914 to 2012.

The record at McNary 2N (1934-2012) needed an extension from 1914 to 1933 (20 years) and the record at Pinetop Fish Hatchery (1944-2012) needed an extension from 1914 to 1943 (30 years). The records were extended by developing and applying regression equations for each month between McNary 2N and Whiteriver 1 Sw and between Pinetop Fish Hatchery and Payson composite record. Eight precipitation sites (Alpine, Gisela, Natural Bridge, Payson composite record, Pinedale, Pleasant Valley Ranger Station/Young composite record, Roosevelt 1 Wnw, and Whiteriver 1 Sw) had the necessary data (early record and common record) that could be used in the regression extensions. Whiteriver 1 Sw and Payson composite records were selected as the predictor (explanatory) variables in the regression extensions because they had the highest correlations with monthly precipitation at McNary 2N and Pinetop Fish Hatchery, respectively.

The equations for estimating monthly precipitation at McNary from 1914 to 1933 were developed by using the common data with Whiteriver 1 Sw from 1934 to 2012. The r^2^ values for regression equations for January-May and September-December ranged from 0.59 to 0.91 and averaged 0.76; the standard errors ranged from 34 to 55 percent and averaged 41 percent. The standard errors are expressed as the percent of the mean value of the response variable (mean monthly precipitation at McNary). The r^2^ values for regression equations for the summer months (June-August) ranged from 0.27 to 0.47 and averaged 0.35; the standard errors ranged from 31 to 99 percent and averaged 57 percent.

The equations for estimating monthly precipitation at Pinetop Fish Hatchery from 1914 to 1943 were developed by using the common data with Payson composite record from 1944 to 2012. The r^2^ values for regression equations for January-May and September-December ranged from 0.49 to 0.84 and averaged 0.69; the standard errors ranged from 33 to 80 percent and averaged 48 percent. The r^2^ values for regression equations for the summer months (June-August) ranged from 0.29 to 0.50 and averaged 0.36; the standard errors ranged from 40 to 86 percent and averaged 55 percent.

Some analyses were done to justify and validate the record extensions at McNary and Pinetop Fish Hatchery. The characteristics and temporal variation of precipitation in the extended records needed to be representative of and similar to the regional precipitation characteristics during the same time periods. Trend analyses and correlation analyses were done on the record-extension period for all sites to validate the record extensions.

Trends in precipitation were tested from 1914 to 1945 for January, April, July, and October at the 8 sites with data for that period (table). The four months were selected to represent the four seasons. No significant trends were found in the monthly data at these sites, including Whiteriver 1 Sw and Payson Ranger Station. Therefore, no trends in the explanatory variables (Whiteriver 1 Sw and Payson composite record) were transferred to the record extensions for McNary and Pinetop fish hatchery. Correlation coefficients were also computed for the four months at all 8 sites for 1914 to 1945 and correlations were high for all sites. The average correlation coefficient was 0.86 for January, 0.76 for April, 0.62 for July, and 0.67 for October. The lack of trends and the high correlations indicate that all the sites were experiencing similar precipitation characteristics over that time period.

The McNary extended record was chosen for regression analyses because it had the highest correlations with monthly flows. Explanatory variables in linear least square regression analysis are assumed to be observed and measured with no error. While the McNary record needed to be extended and therefore was not observed across entire record, the extensions were short and we assumed the natural variability of the other weather stations used for these extensions were transferred and captured to McNary record.
